# FAK signaling suppression by OCT4-ITGA6 mediates the effectively removal of residual pluripotent stem cells and enhances application safety

**DOI:** 10.7150/thno.111198

**Published:** 2025-06-12

**Authors:** Wenpeng Song, Jian Wang, Shixin Gong, Xiaoyan Wang, Junji Xu, Ruiqing Wu, Zongmin Jiang, Huiyuan Zhang, Lida Wu, Yilong Wang, Yingying Su, Hao Wang, Yuchun Gu

**Affiliations:** 1 Department of Stomatology, Beijing Tiantan Hospital, Capital Medical University, Beijing, China.; 2 Translational Medicine Research Group (TMRG), Aston Medical School, Aston University, Birmingham, UK.; 3 Molecular Pharmacology Laboratory, Institute of Molecular Medicine, Peking University, Beijing, China.; 4 Research and Development Department, Allife Medicine Inc., Beijing, China.; 5 Beijing Laboratory of Oral Health, Capital Medical University School of Stomatology, Beijing, China.; 6 College of Stomatology, Chongqing Medical University, Chongqing, China.; 7 Department of Neurology, Beijing Tiantan Hospital, Capital Medical University, Beijing, China.

**Keywords:** Pluripotent stem cells, Teratoma, balanced salt solution, integrin, focal adhesion kinase

## Abstract

**Rationale:** Pluripotent stem cells (PSCs) serve as a critical source of seed cells for regenerative therapies due to their unlimited proliferative capacity and ability to differentiate into all three germ layers. Despite their potential, the risk of teratoma formation caused by residual PSCs within differentiated cell populations poses a significant barrier to clinical applications. This study aims to develop a novel strategy to selectively remove residual PSCs while preserving the safety and functionality of PSC-derived differentiated cells (iDCs).

**Methods:** The calcium- and magnesium-free balanced salt solution (BSS(Ca-Mg-)) was employed to selectively target PSCs in a co-culture system comprising PSCs and four types of iDCs. The effect of BSS(Ca-Mg-) treatment on teratoma formation was evaluated in immunodeficient mice following cell transplantation. Comparative analysis and gene knockdown experiments were conducted to explore the molecular mechanisms underlying the differential response of PSCs and iDCs to BSS(Ca-Mg-), focusing on FAK signaling and its interaction with OCT4 and ITGA6.

**Results:** The BSS(Ca-Mg-) treatment effectively induced the detachment of PSCs in the co-culture system without disrupting iDC adhesion. *In vivo* experiments confirmed that cells treated with BSS(Ca-Mg-) did not form teratomas upon implantation into immunodeficient mice. Mechanistic studies revealed that PSCs exhibit lower activation of FAK signaling compared to iDCs, contributing to their selective detachment. Additionally, OCT4 and ITGA6 were found to maintain each other's protein expression, forming a feedback loop that suppressed FAK signaling, while FAK suppression further enhanced OCT4 expression.

**Conclusions:** The study presents a safe, effective, and cost-efficient method for the selective removal of residual PSCs. This approach enhances existing safety measures for iDC applications, improving the clinical feasibility of iDC-based cell therapies.

## Introduction

Pluripotent stem cells (PSCs), renowned for their capacity for unlimited self-renewal and differentiation into all three germ layers, have emerged as a promising cell source for regenerative therapies aimed at treating a wide range of diseases and injuries 1-3. Since the groundbreaking work by James Thomson's group in 1998, which first introduced human embryonic stem cells (ESCs) 4, two main types of human PSCs (induced pluripotent stem cells (iPSCs) and ESCs), have been extensively studied for potential clinical applications 5-7. To date, more than 90 clinical trials involving PSCs have been registered, exploring their therapeutic potential in treating conditions such as heart failure, retinal degenerative disorders, and acute ischemic stroke.

Currently, PSC-based cell therapies are generating considerable excitement. However, significant challenges remain regarding their clinical application, including ethical concerns related to embryo use, tumorigenicity, immunogenicity, and cellular heterogeneity. Among these, teratoma formation is one of the most critical risks associated with PSC transplantation 1, 8-10. A major advantage of PSCs is their capacity for unlimited expansion, which enables the production of large quantities of human cells for therapeutic use. However, this same proliferative capacity poses a risk of tumor development if the cells continue to divide post-transplantation. Even a small number of residual PSCs in the transplanted cell population can potentially lead to teratoma formation. For instance, a 2022 case report documented the formation of an immature teratoma following the injection of PSC-derived pancreatic β-cells 11. As such, the effective detection and removal of residual PSCs in PSC-derived cell therapies are essential to enhancing the safety of these treatments and overcoming barriers to their broader clinical application.

Several strategies have been explored to reduce residual PSCs from differentiated cell populations to reduce the risk of teratoma formation 12, 13. One promising approach leverages the differential sensitivity of PSCs and PSC-induced differentiated cells (iDCs) to apoptosis-inducing agents, enabling the selective removal of PSCs using small molecules. In 2004, Jeong *et al.* 14 demonstrated that S18 (N-oleoyl serinol) effectively induced apoptosis in murine embryoid body (EB) cells, selectively removing OCT4-positive stem cells while enriching Nestin-positive neural precursor cells. This strategy not only prevented teratoma formation but also promoted neural differentiation in implanted cells in mouse models 14. Since then, various small molecules or proteins, including Bee Venom, L-Alanine, PluriSIn#1, phospho-D-peptides, clostridium perfringens enterotoxin, lectin-toxin fusion protein, and JC011, have shown potential for clearing residual PSCs 15-21. Although small-molecule/protein approaches are effective and relatively simple, they present challenges in ensuring the functional integrity and safety of all PSC-derived cell types *in vivo*, given the diverse biological properties of these cells 12. In addition to small molecules/proteins, other methods like gene editing strategies that introduce suicide genes or miRNA switches, metabolic regulation, as well as antibody-based selective removal, have gained attention in recent years 12, 13, 22-27. However, the introduction of foreign genes into PSCs carries additional risks, such as genetic mutations, and issues related to reagent residues further complicate their clinical application.

Some biophysical approaches also hold potential for the removal of residual PSCs. Two studies from the same research group successfully achieved effective separation of PSCs from differentiated cells using dielectrophoresis and controlled fluid flow, without the need for fluorescent dyes or magnetic antibodies 28, 29. Although the primary objective of these studies was different, the principle underlying this method suggests the possibility of effectively removing residual PSCs by selectively collecting differentiated cells 28, 29. However, the researchers did not further investigate the characteristics of the treated differentiated cells, and whether this method affects their properties remains to be verified. Visible light and irradiation have also been explored for the removal of residual PSCs. A study by Cho *et al.* 30 demonstrated that CDy1, a fluorescent probe specifically targeting PSCs, can selectively induce PSC death upon visible light exposure, while leaving differentiated endothelial cells unaffected. Takeda *et al.* 31 demonstrated that X-ray irradiation can effectively remove residual PSCs from PSC-derived cardiomyocytes. Furthermore, Chen *et al.* 32 leveraged size differences between suspended cells and utilized an inertial microfluidic-based device to achieve label-free, high-throughput separation of PSCs from PSC-derived spinal cord progenitor cells. However, challenges such as potential probe residues and whether these approaches can achieve harmless PSC removal in other PSC-derived differentiated cell types remain unexplored. Given the limitations of current strategies for residual PSC clearance, there is a pressing need to develop novel or alternative approaches that can effectively and selectively remove PSCs without compromising the characteristics of iDCs.

The adhesion of adherent cells (such as mesenchymal stem cells or fibroblasts) is primarily mediated by the integrin receptor family 33-35. These receptors can sense both chemical and mechanical properties of the extracellular microenvironment and generate functional responses that regulate cellular behavior 33. The activation of integrins depends on the participation of divalent cations, including Ca²⁺, Mg²⁺, and Mn²⁺ 36. When the concentration of divalent cations in the surrounding environment changes, the structure of integrins and their interactions with ligands are altered, thereby influencing the cell adhesion process 37, 38. We serendipitously discovered that treatment with calcium- and magnesium-free balanced salt solution (BSS(Ca-Mg-)) leads to a rapid loss of adhesion in PSCs, while multiple iDCs demonstrate greater resistance to the treatment. Leveraging the observed differences in cellular responses, this study employs iPSCs, extended pluripotent stem cells (EPSCs), and iDCs as models to investigate the efficacy of BSS(Ca-Mg-) in clearing residual PSCs from differentiated cells. Additionally, it investigates the mechanisms underlying the differential responses of PSCs and iDCs to BSS(Ca-Mg-) treatment. The study hypothesizes that BSS(Ca-Mg-) can rapidly and cost-effectively remove residual PSCs without causing additional damage to the cells. This approach could mitigate concerns surrounding the clinical use of iDCs and potentially accelerate progress in PSC-based therapies in both research and clinical settings.

## Methods and Materials

### PSCs and Human Umbilical Vein Endothelial Cells (HUVECs) culture

The pluripotent stem cell lines were provided by Allife Medicine Inc. (http://www.allifetech.com/), including iPSCs-001-5, iPSCs-006-1, EPSCs-001-5, and EPSCs-006-1. TeSR™-E8™ Medium (E8, #05990, STEMCELL Technologies, British Columbia, Canada) was used to maintain the culture of iPSCs. The maintenance medium for EPSCs consisted of DMEM/F12 (11330032, Gibco, California, USA) and Neurobasal (21103049, Gibco, California, USA) (1:1), supplemented with 1% GlutaMAX (35050061, Gibco, California, USA), 1% NEAA (11140050, Gibco, California, USA), 0.1mM β-Mercaptoethanol (21985023, Merck, Hesse, Germany), 3% KSR (10828028, Gibco, California, USA), 0.5% N2 (17502048, Gibco, California, USA), 1% B27 (12587010, Gibco, California, USA), 1% ITS-X (51500056, Gibco, California, USA), 100μg/mL L-AA-pi (a4544, Sigma-Aldrich, Missouri, USA), 2 μM (S)-(+)-Dimethindene maleate (HY-107647, MedChemExpress, New Jersey, USA), 10 ng/mL LIF (30005, Peprotech, New Jersey, USA), 40ng/mL Activin A (#78001.3, STEMCELL Technologies, British Columbia, Canada), 2 μM Minocycline hydrochloride (HY-17412, MedChemExpress, New Jersey, USA), 10 μM Trolox (HY-101445, MedChemExpress, New Jersey, USA), 1 μM CHIR99021 (S1263, Selleck, Texas, USA), 5 μM Y-27632 (ab120129, Abcam, Cambridge, UK), 2 μM XAV939 (ab120897, Abcam, Cambridge, UK), and 1 μM GSK126 (S7061, Selleck, Texas, USA).

During the maintenance culture, the medium was changed daily. When the PSCs reached 80% confluence, the cells were passaged. Cells were digested with 5μM EDTA at 37 °C for 5 min and then seeded onto Matrigel-coated dishes. For iPSC passaging, an additional 10μM Y27632 was added to the TeSR™-E8™ Medium, which was removed 24 hours after seeding. In contrast, no additional Y27632 was required for EPSC passaging. Finally, only PSCs with less than 35 passages were used for subsequent experiments.

When assessing the pluripotency of PSCs, HUVECs were used as a negative control. HUVECs were purchased from ScienCell Research Laboratories (Catalog #8000; Sciencell, CA, USA) and maintained in endothelial cell medium (ECM, Catalog #1001; Sciencell, CA, USA). HUVECs at passages 3 to 5 were used for subsequent experiments.

### Differentiation procedures of iPSCs into mesenchymal stem cells (iMSCs), fibrochondrocytes (iFCs), osteoblasts (iOBs), endothelial progenitor cells (EPCs), neuroectoderm, and mesendoderm

To harvest cells for co-culture experiments, iPSCs were directed to differentiate into iMSCs, iFCs, iOBs, and iEPCs, respectively.

**iMSCs:** The method similar to that used in previously published studies was employed for the induction of iMSC differentiation 39. Briefly, iPSCs were maintained in TeSR™-E6 medium (E6, #05946, STEMCELL Technologies, British Columbia, Canada) containing 10 ng/mL BMP4 (ab87063, Abcam, Cambridge, UK), 4 μM SB431542 (ab120163, Abcam, Cambridge, UK), and 0.1 μM PD173074 for 5 days. After digestion, the cells were passaged at a density of 4 × 10^4^ cells/cm^2^ onto Matrigel-coated 6-well plates and further cultured in αMEM (11900073, Gibco, California, USA) containing 5% UltraGRO (HPCFDCRL50, Helios, Berlin, Germany), designated as passage 1 (P1). Cells were passaged every 6 days, and passages P3-P8 were used for subsequent experiments.

**iFCs:** The iFCs were induced from iPSCs according to the protocol of Kaji *et al.* 40. In short, iPSCs were cultured in TeSR™-E8™ Medium supplemented with 500 nM LDN-193189 (S2618, Selleck, Texas, USA) for 2 days to form EBs. The induction was then continued for 4 days with DMEM (11320033, Gibco, California, USA) supplemented with 500 nM LDN-193189, 5 μM CHIR99021 and 100 nM AGN 193109 (HY-U00449, MedChemExpress, New Jersey, USA). The culture was then incubated for another 4 days, and the medium was prepared using DMEM supplemented with 10 ng/mL TGFβ1 (CA32002, Cellapybio, Beijing, China), 100 nM SAG (S7779, Selleck, Texas, USA), 10 ng/mL FGF2 (AF-100-18B, Peprotech, New Jersey, USA), and 100 nM AGN 193109.

**iOBs:** For the induction of osteogenic lineages from iPSCs, this study follows the protocol in the article by Kawai *et al.* 41. The iPSCs were first cultured in an environment containing 20% mTeSR1 and 80% osteogenic induction medium for 24 hours. Cultures were started from the second day using 100% osteogenic induction medium and changed on the fourth and seventh days. The osteogenic induction medium consisted of KnockOut DMEM (10829018, Gibco, California, USA), 20% FBS (Cellmax, SA211.01, Taiwan, China), 1% GlutaMAX, 10 mM glycerol-2-phosphate (G9422, Sigma-Aldrich, Missouri, USA), 1 nM dexamethasone (D4902, Sigma-Aldrich, Missouri, USA), 0.1 mM β-Mercaptoethanol, 50 μg/mL 2-Phospho-L-ascorbic acid trisodium salt (49752, Sigma-Aldrich, Missouri, USA), 1% NEAA, 10 μM Y27632, and 1 μM retinoic acid (R2625, Sigma-Aldrich, Missouri, USA).

**iEPC:** The iPSCs were differentiated into EPCs in 2D monolayer-based serum-free cultures using a published procedure 42. In brief, the differentiation of iEPCs was divided into two phases, the first phase (3 days) from iPSCs to mesoderm and the second phase (2 days) to further differentiate into iEPCs. The first phase medium was DMEM/F12 supplemented with N2, B27, β-Mercaptoethanol, 25 ng/mL BMP4, 10 μM CHIR99021, 50 ng/mL Activin A (added only on the first day). Stage II medium was selected as StemPro-34 SFM medium supplemented with 50 ng/mL VEGFA, 10 μM SB431542, and Forskolin.

**Neuroectoderm:** The iPSCs were seeded at a density of 2×10^4^ cells/cm^2^ in E8 medium supplemented with 10 µM Y27632 onto Matrigel-coated 6-well plates. After 24 hours, the medium was replaced with neuroectoderm differentiation medium, which was refreshed daily for 7 days. The neuroectoderm differentiation medium consisted of DMEM/F12 (11330032, Gibco, California, USA) supplemented with 2% B27, 1% N2, 1% NEAA, 1% GlutaMAX, 25 µg/mL Insulin (abs42019847, Absin Bioscience, Shanghai, China), 10 µM SB431542, 100 nM LDN193189, 100 nM retinoic acid, and 0.1 µM β-Mercaptoethanol.

**Mesendoderm:** When iPSCs reached 70% confluence in Matrigel-coated 6-well plates, mesendoderm induction was initiated. The mesendoderm induction medium consisted of IMDM/F12 (1:1, 12440053/ 11765054, Gibco, California, USA) supplemented with 0.5% BSA (A1933, Sigma-Aldrich, Missouri, USA), 550 nM thioglycerin, 1% Chemically Defined Lipid Concentrate (CDLC, 11905031, Gibco, California, USA), 1% penicillin/streptomycin (15140122, Gibco, California, USA), 100 ng/mL Activin A, and 3 µM CHIR99021. The medium was refreshed daily for 5 days.

### Establishment of a co-culture system for iPSCs and their differentiated cells

In this study, the co-culture system of PSCs and their differentiated cells was used to simulate the presence of residual pluripotent stem cells in a realistic differentiation environment. First, PSCs were digested with 5μM EDTA and seeded at different densities (3×10^4^/cm^2^ and 9×10^4^/cm^2^) onto Matrigel-coated 6-well plates using TeSR™-E8™ Medium supplemented with 10 μM Y27632. Four hours later, the medium was replaced with TeSR™-E8™ Medium and the cells were cultured for an additional 20 hours.

For the differentiated cells, they were digested with TrypLE at 37 °C for 5 min. After centrifugation, the cells were resuspended in their respective maintenance media and seeded at different densities onto the 6-well plates containing adherent PSCs. Differentiated cells were added at a density of 9x10^4^/cm^2^ to the 6-well plates with PSCs at a density of 3x10^4^/cm^2^. Conversely, for PSCs at a density of 9 x 10^4^/cm^2^, the differentiated cells were added at a density of 3x10^4^/cm^2^. To minimize the impact of the differentiation maintenance media on the pluripotency of PSCs, the co-culture system was considered established 8 hours after the addition of the differentiated cells (when the differentiated cells had adhered). Subsequent experiments were then carried out.

### Tri-lineage differentiation of iMSCs

The tri-lineage differentiation potential of iMSCs was assessed by inducing adipogenic, osteogenic, and chondrogenic differentiation. For osteogenic and adipogenic differentiation, the MesenCult™ Osteogenic Differentiation Kit (Catalog # 05465, STEMCELL Technologies, British Columbia, Canada) and the MesenCult™ Adipogenic Differentiation Kit (Catalog # 05412, STEMCELL Technologies, British Columbia, Canada) were used, respectively. Briefly, when iMSCs cultured in 6-well plates reached 90% confluency, the medium was replaced with osteogenic or adipogenic induction medium. The cells were maintained in the induction medium for 14 days, with the medium being refreshed every 3 days.

For chondrogenic differentiation, a 3D pellet culture system was utilized. Briefly, 1 × 10^5^ iMSCs were resuspended in 0.5 mL of MesenCult™-ACF Chondrogenic Differentiation Medium (Catalog # 05455, STEMCELL Technologies, British Columbia, Canada) in a 15 mL polypropylene tube. The cell suspension was centrifuged at 300 × g for 5 min at room temperature to form a pellet. The caps of the tubes were gently loosened, and the cells were cultured at 37°C in a humidified atmosphere with 5% CO2 for 3 days. After 3 days, 0.5 mL of Chondrogenic Differentiation Medium was added to each tube, and the medium was partially changed every 3 days thereafter. The chondrogenic pellets were harvested on day 21 for further analysis.

### Colony formation assay

The colony formation assay was conducted to evaluate the self-renewal potential of iMSCs. The iMSCs were trypsinized to create a single-cell suspension and then seeded in 6-well plates at a low density of 500 cells per well in 2 mL of MSCM. The cells were cultured under standard conditions (37 °C, 5% CO2) for 14 days to allow colony formation. During this period, the medium was refreshed every 2 days. At the end of the incubation period, when visible colonies had formed, the medium was gently aspirated, and the cells were washed twice with PBS. The colonies were then fixed with 4% paraformaldehyde for 10 min and subsequently stained with 0.1% crystal violet solution for 30 min at room temperature. The number of colonies containing at least 50 cells was counted manually. The colony formation efficiency was calculated as the percentage of seeded cells that formed colonies.



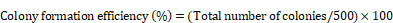



### Alizarin red staining

Alizarin Red staining was performed to assess calcium deposition as a marker of osteogenic differentiation. The iMSCs treated with DPBS(Ca-Mg-) and the control iMSCs underwent osteogenic differentiation as described earlier. At the end of the differentiation period, the medium was aspirated, and the cells were gently washed twice with PBS. The cells were then fixed with 4% paraformaldehyde and washed twice to remove any residual fixative. Afterward, the cells were stained with 0.2% Alizarin Red S solution (C0140, Beyotime Biotechnology, Jiangsu, China) for 30 min at room temperature.

### Oil red O staining

To evaluate lipid accumulation as a marker of adipogenic differentiation, Oil Red O staining was performed. The iMSCs treated with DPBS(Ca-Mg-) and the control iMSCs underwent adipogenic differentiation as described earlier. Upon completion of the differentiation period, the culture medium was carefully removed, and the cells were rinsed twice with PBS. The Oil Red O Stain Kit (G1262, Solarbio LIFE SCIENCES, Beijing, China) was used to assess the adipogenic differentiation potential of the cells, following the manufacturer's instructions.

### Alcian Blue staining

To detect glycosaminoglycan (GAG) accumulation in chondrogenic pellets derived from iMSCs, an Alcian Blue staining procedure was carried out. The pellets formed by DPBS(Ca-Mg-)-treated iMSCs and control iMSCs, as described above, were collected. The fixed pellets were cryoprotected by soaking them sequentially in 15% and 30% sucrose solutions (S8271, Solarbio LIFE SCIENCES, Beijing, China) in PBS at 4 °C until they sank, indicating full saturation. Subsequently, the pellets were embedded in optimal cutting temperature (OCT) compound (4583, Sakura Finetek, California, USA), rapidly frozen, and sectioned into 20 µm slices using a cryostat. The sections were mounted on glass slides. Following the manufacturer's instructions, Alcian Blue staining was performed using the Alcian Blue Stain Kit (G1560, Solarbio LIFE SCIENCES, Beijing, China).

### Wound healing assay

The wound healing assay was conducted to evaluate the migratory capacity of iMSCs and those treated with DPBS(Ca-Mg-). The iMSCs were plated in 6-well plates and cultured until reaching 90-100% confluency in complete growth medium. One group of iMSCs was treated with DPBS(Ca-Mg-) at 37 °C for 30 min, followed by replacement with MSCM for continued culture over 24 hours. The control group was directly replaced with MSCM.

Following treatment, a sterile 200 µL pipette tip was used to create a straight scratch (wound) across the cell monolayer. The wells were then gently washed twice with PBS to remove any detached cells and debris. The cells were subsequently cultured in αMEM to minimize proliferation and emphasize migration. Images of the wound were captured immediately after scratching (0 hours) and at subsequent intervals (3 hours, 6 hours, and 72 hours). Wound closure was quantified by comparing the wound width at each time point to the initial width at 0 hours.

### Cell Counting Kit-8 (CCK8) assay

The CCK-8 assay was conducted to assess the proliferation and viability of iMSCs. The iMSCs were seeded into 96-well plates at a density of 5 × 10^3^ cells per well in 100 µL of MSCM. After allowing the cells to adhere overnight (marked as 0h), a portion of the iMSCs was treated with DPBS(Ca-Mg-) at 37 °C for 30 min, while the rest remained untreated. The CCK-8 assay was then performed at 0, 24, 48, and 72 hours. At each indicated time point, 10 µL of CCK-8 solution (C0037, Beyotime Biotechnology, Jiangsu, China) was added to each well, and the plates were incubated at 37 °C with 5% CO2 for 1 hour. The absorbance was measured at 450 nm using a microplate reader.

### Crystal violet staining

Following different treatments, the culture medium was removed, and the cells were gently washed twice with PBS to remove any residual medium. Cells were then fixed with 4% paraformaldehyde for 20 min at room temperature. Following fixation, the cells were washed three times with PBS. Following the washing steps, the cells were stained with 0.1% (w/v) crystal violet solution (110703008, BKMAN, Hunan, China/C0121, Beyotime Biotechnology, Jiangsu, China) at room temperature for 30 min. The cells were then gently rinsed with PBS to remove excess dye. Photographs were taken to record the results.

### Trypan blue exclusion assay

To assess cell viability, the trypan blue exclusion assay was performed. Cells were harvested by trypsinization and resuspended in an appropriate volume of complete culture medium. A 10 µL aliquot of the cell suspension was mixed with 10 µL of 0.4% trypan blue solution (T8154, Sigma-Aldrich, Missouri, USA) and incubated for 3 min at room temperature. Following incubation, 10 µL of the trypan blue-cell mixture was loaded into a hemocytometer, and both viable (unstained) and non-viable (blue-stained) cells were counted under a light microscope. Cell viability was calculated as the percentage of viable cells relative to the total number of cells counted. The formula used was:



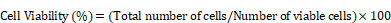



### Alkaline phosphatase (ALP) staining

The undifferentiated PSCs express high levels of ALP, and ALP staining was used to identify PSCs applied in this study. The PSCs were seeded in 6-well plates or 12-well plates and cultured until they reached the desired confluency. The culture medium was removed, and the cells were gently washed twice with PBS. Cells were then fixed with 4% PFA for 10 min at room temperature. After fixation, cells were washed three times with PBS and incubated with the ALP staining solution (C3206, Beyotime Biotechnology, Jiangsu, China) at room temperature for 2 hours in the dark. After the incubation, the staining solution was removed, and the cells were washed three times with distilled water to stop the reaction.

### EGFP, shOCT4, and shITGA6 lentiviral vector transduction

iPSCs were transduced with lentiviruses carrying EGFP, shOCT4, and shITGA6 to facilitate tracking in co-culture systems and to study the signaling and phenotypic changes following the knockdown of OCT4 and ITGA6 in iPSCs. According to the manufacturer's instructions, PEI Prime™ Powder (PRIME-P100-1G, SEROCHEM, Guangdong, China) was used to co-transfect HEK293T cells with the packaging plasmids pRSV-Rev, pVSV-G, pMD2.G, and the target plasmids to produce lentiviral particles. Forty-eight hours post-transfection, the culture supernatant containing lentiviral particles was collected, filtered through a 0.45 µm membrane, and subsequently concentrated using polyethylene glycol (PEG) 8000 (V900156, Sigma-Aldrich, Missouri, USA).

Lentiviral transduction was performed when the cells reached approximately 70% confluence. The iPSCs were incubated in TeSR™-E8™ Medium containing 10 µg/mL Polybrene (TR-1003, Sigma-Aldrich, Missouri, USA) and concentrated lentiviral supernatant. After 24 hours, the medium was replaced with fresh TeSR™-E8™ Medium. To select for stably transduced cells, 2 µg/mL puromycin (A1113803, Sigma-Aldrich, Missouri, USA) was added to the medium, and selection was maintained for 3 days. The transduction efficiency of EGFP was subsequently assessed using immunofluorescence. The knockdown efficiency of shOCT4 and shITGA6 proteins was verified by qPCR and immunofluorescence. Once the sequences are validated for efficacy, they will be used for subsequent functional and expression studies. The validated shRNA sequences will be transfected using the aforementioned methods, followed by cell selection through puromycin treatment for three days. The cells after puromycin selection will be directly used for subsequent qPCR analysis and immunofluorescence staining.

The following shRNA sequences were used:

Human OCT4: 5'-GTGGATGTGGTCCGAGTGTGGTTCAAGAGACCACACTCGGACCACATCCTTTTTT-3';

Human ITGA6-1: 5'-ACCGGTGCACATTTCTAGAGGAATACTCGAGTATTCCTCTAGAAATGTGCTTTTTTGAATTC-3';

Human ITGA6-2: 5'-ACCGGTGGATATGCCTCCAGGTTAACTCGAGTTAACCTGGAGGCATATCCTTTTTTGAATTC-3';

Human ITGA6-3: 5'-ACCGGTTGATAGAGATGGAGAAGTTCTCGAGAACTTCTCCATCTCTATCATTTTTTGAATTC-3'.

### ITGA6 function blocking

In addition to using shRNA to knockdown ITGA6, this study also employed ITGA6 function blocking to investigate ITGA6 and its downstream effects in iPSCs. First, iPSCs were cultured on Matrigel-coated plates. When the confluence reached 70%, 40 µg/mL ITGA6 function-blocking antibody (GoH3, 14-0495-82, Invitrogen, Carlsbad, California, USA) was added to the medium and incubated for 24 hours to block ITGA6. Subsequent analyses were performed to assess the resistance of iPSCs (with or without ITGA6 function blocking) to DPBS (Ca-Mg-) as well as FAK signaling status using immunofluorescence.

### EB formation and assessment

EBs are three-dimensional structures that form spontaneously from PSCs *in vitro*, containing cells from the three germ layers (endoderm, mesoderm, and ectoderm), which is the critical indicator of the differentiation potential of PSCs. To form EBs, iPSCs were cultured in low-adhesion 6-well plates (10× 10^5^ iPSCs/well, 3471, Corning, New York, USA) for 24 hours in TeSR™-E8™ Medium supplemented with 10 µM Y27632. Following this, the cells were cultured for an additional 12 days in EB formation medium, with the medium being changed every 2 days. The EB formation medium consisted of DMEM/F12 supplemented with 15% KSR, 1% NEAA, 1% GlutaMAX, and 0.1 mM β-Mercaptoethanol. After the EBs were formed, they were transferred to Matrigel-coated 6-well plates and further cultured for 2 days in EB formation medium. The gene and protein expression levels of the three germ layer markers were assessed using qPCR and immunofluorescence.

### RNA isolation, reverse transcription and qPCR

The total RNA was extracted using TRIzol^TM^ reagent (10296010, Invitrogen, Carlsbad, California, USA). The RNA with A260/280 value between 1.8-2.1 determined by nanodrop 2000c were used for further study. Reverse transcription was performed applying TransScript® One-Step gDNA Removal and cDNA Synthesis SuperMix (#AT311-02, Transgen Biotech, Beijing, China) according to the manufacturer's instructions.

The TransStart^®^ Green qPCR SuperMix (#AQ131-01, Transgen Biotech, Beijing, China) and the Roche light-cycle 480 real-time PCR system were applied for the mRNA expression levels measurement. GAPDH was used as the internal reference. The relative expression of transcripts was calculated using the formula fold = 2^-ΔΔCT^. The primer sequences were shown in **Table S1**.

### Western blotting

When the Western blotting experiment is required, the cells were first washed twice with cold PBS in the culture dish. Cells were lysed using RIPA lysis buffer (P1013B, Beyotime Biotechnology, Jiangsu, China) supplemented with 1 mM PMSF (ST506, Beyotime Biotechnology, Jiangsu, China) and a 1× Protease and Phosphatase Inhibitor Cocktail (P1045, Beyotime Biotechnology, Jiangsu, China). The dish was incubated on ice for 30 min, with occasional gentle agitation to promote lysis. The lysate was collected using a cell scraper and transferred to a centrifuge tube. After centrifuging at 14,000 × g for 15 min at 4 °C, the supernatant was collected. The loading buffer (P0015L, Beyotime Biotechnology, Jiangsu, China) was added to the protein lysate at a ratio of 1:4 and heated at 95 °C for 8 min.

Equal amounts of protein samples were separated by SDS-PAGE and transferred onto polyvinylidene fluoride (PVDF) membrane. The membranes were blocked with 5% non-fat milk (P0216, Beyotime Biotechnology, Jiangsu, China) or BSA (ST025, Beyotime Biotechnology, Jiangsu, China) in 1× TBST (ST673, Beyotime Biotechnology, Jiangsu, China) for 1 hour at room temperature. Then, the membrane was incubated with the primary antibody overnight at 4 °C. After washing with TBST, the membranes were incubated with corresponding secondary antibody at room temperature for 1 hour. Protein bands were visualized using the Syngene G-Box imaging system (Syngene, Cambridge, UK) and ECL Plus Western Blotting Substrate (Catalog #32132; ThermoFisher Scientific, Massachusetts, USA), and quantified using ImageJ software (Fiji). The information of primary antibody applied in this part was listed at **Table S2**.

### Immunofluorescence (IF) staining and fluorescence detection

For immunofluorescence, cells were fixed in PFA for 15 min at room temperature. For non-membrane proteins, cells need to be permeabilized for an additional 20 min with 0.2% Triton-X-100 (T8200, Solarbio LIFE SCIENCES, Beijing, China). Then, cells were blocked in 5% BSA for 30 min and labeled for 4 hours at room temperature or overnight at 4 °C with various primary antibodies. Cells were incubated with fluorescent secondary antibodies for 60 min at room temperature, followed by Nuclei counterstaining with DAPI (C0060, Solarbio^®^ LIFE SCIENCES, Beijing, China). For observation of live cells transfected with EGFP, fixation and antibody incubation are not required. Images were acquired using OLYMPUS confocal microscope.

### Phalloidin staining

Phalloidin staining was performed to visualize filamentous actin (F-actin) structures in cultured cells. iPSCs and iMSCs were cultured in 12-well plates and then treated with calcium- and magnesium-free DPBS, calcium- and magnesium-free PBS, calcium- and magnesium-containing DPBS, and E8/MSCM, respectively. The cells were gently washed twice with PBS. They were then fixed with 4% paraformaldehyde (PFA, DF0135, Leagene Biotechnology, Beijing, China) in PBS for 15 min at room temperature. Following fixation, the cells were washed three times with PBS and permeabilized with 0.1% Triton X-100 in PBS for 5 min. After permeabilization, the cells were washed again three times with PBS. To stain F-actin, the cells were incubated with Phalloidin conjugated to Alexa Fluor 594 (1:100, C2205S, Beyotime Biotechnology, Jiangsu, China) for 30 min at room temperature in the dark. After staining, the cells were washed three times with PBS to remove excess Phalloidin. Then, as with immunofluorescence, the cell nuclei were stained with DAPI and observed and recorded using an OLYMPUS confocal microscope.

### Flow cytometry

Flow cytometry was performed to assess the expression of cell markers and cell cycle distribution. Cells were harvested by TrypLE, washed twice with cold phosphate-buffered saline (PBS), and fixed in 4% PFA for 20 min at room temperature. For surface marker analysis, cells were incubated with fluorochrome-conjugated antibodies specific for the target markers for 30 min at room temperature in the dark. After incubation, cells were washed twice with PBS to remove unbound antibodies and resuspended in 500 µL of PBS. The information of primary antibody applied in this part was listed at **Table S2**. For cells transfected with EGFP, the cells were resuspended using PBS containing 0.4% BSA instead of fixing them. The cells were then detected in the same way as the other cells treated with antibodies.

For cell cycle analysis, cells were fixed in 70% ethanol at -20 °C for at least 2 hours, followed by incubation with 50 µg/mL propidium iodide (PI) and 100 µg/mL RNase A (Cell Cycle and Apoptosis Analysis Kit, C1052, Beyotime Biotechnology, Jiangsu, China) in the dark for 30 min at room temperature.

Data acquisition was performed using a BD FACSCanto II flow cytometer (BD Biosciences, California, USA), and at least 3000 events were collected for each sample. Specific fluorescence channels were used to assess marker expression or DNA content. Results were expressed as percentages of positive cells for surface markers or as distributions across different phases of the cell cycle (G0/G1, S, and G2/M).

### RNA-sequencing (RNA-seq)

Total RNA was extracted from cells using the TRIzol^TM^ reagent according to the manufacturer's instructions. The RNA quality was assessed using the Agilent 2100 Bioanalyzer (Agilent Technologies, CA, USA). To construct RNA-seq libraries, mRNA was enriched using Oligo dT-attached magnetic beads, fragmented, and reverse-transcribed into cDNA.

The cDNA was then ligated to sequencing adapters, and PCR amplification was performed to enrich the libraries. After the library construction was completed, the library was initially quantified using the Qubit 2.0 Fluorometer (ThermoFisher Scientific, Massachusetts, USA) and then diluted to 1.5 ng/µL. The insert size of the library was subsequently analyzed using the Agilent 2100 Bioanalyzer. Once the insert size met the expected criteria, the effective concentration of the library was accurately determined using qRT-PCR (with the effective concentration being higher than 1.5 nM) to ensure the quality of the library. After the library passed quality control, different libraries were pooled according to their effective concentration and the required target data output for sequencing. The pooled libraries were then subjected to Illumina sequencing.

Differentially expressed genes (DEGs) were identified from RNA-seq data using DESeq2 with a log2|FC|>1 and adjusted p-value < 0.05. The DEGs were divided into upregulated and downregulated groups based on their log2FC values. To gain insights into the biological processes, molecular functions, and cellular components associated with these DEGs, Gene Ontology (GO) enrichment analysis was performed using the clusterProfiler package in R. GO terms with an adjusted p-value < 0.05 were considered significantly enriched. Similarly, KEGG pathway enrichment analysis was conducted using clusterProfiler to identify significantly enriched pathways in the upregulated and downregulated DEGs. The KEGG pathways with an adjusted p-value < 0.05 were considered significantly enriched.

In addition to the individual enrichment analyses, Gene Set Enrichment Analysis (GSEA) was conducted on the entire gene expression dataset to assess the enrichment of predefined gene sets across the ranked list of genes. GSEA was performed by https://www.bioinformatics.com.cn, an online platform for data analysis and visualization. The normalized enrichment score (NES) was calculated for each gene set.

### Chromatin Immunoprecipitation-sequencing (CHIP-seq)

ChIP-seq was performed to investigate the binding of specific transcription factors (OCT4) across the genome. Cells were cross-linked with 1% formaldehyde for 10 min at room temperature, followed by quenching with 125 mM glycine. The cells were then washed with cold PBS, harvested, and lysed in lysis buffer containing protease inhibitors. The chromatin was sheared to an average fragment size of 200-400 bp using a Covaris S220 (Covaris, Massachusetts, USA). The sheared chromatin was incubated overnight at 4 °C with protein A/G magnetic beads pre-bound to specific antibodies against the target protein (OCT4). After immunoprecipitation, the beads were washed, and the bound DNA-protein complexes were eluted and reverse cross-linked. For ChIP-seq library preparation, The DNA fragments were end-repaired and A-tailed. Then, the DNA fragments with A tail were ligated with sequencing adaptors. The final DNA library was obtained after size selection and PCR amplification. Libraries were analyzed for size distribution by Agilent 5400 system (Agilent, California, USA) and quantified by qPCR (1.5nM).

After library quality control, different libraries are pooled according to their effective concentrations and the required amount of data for sequencing on the Illumina platform, generating paired-end reads of 150 bp. The basic principle of sequencing is synthesis while sequencing. In the sequencing flow cell, four fluorescently labeled dNTPs, DNA polymerase, and adapter primers are added for amplification. With each complementary strand extension in a sequencing cluster, the incorporation of a fluorescently labeled dNTP releases corresponding fluorescence. The sequencing instrument captures the fluorescence signals, and computer software converts these signals into sequencing peaks, thus obtaining the sequence information of the target fragments.

For the processing of OCT4 ChIP-seq data, raw reads were trimmed with Trim-Galore software (version 0.4.5). Trimmed reads were mapped to the human (Homo sapiens) hg38 genome obtained from UCSC genome browser database using Bowtie2 (version 2.5.4). Duplicated reads were discarded by MarkDuplicates.jar program in Picard tools (version 1.119). Then, reads were sorted with SAMtools (version 1.2.0). MACS2 (version 1.1) was used to call peak with the parameters set to '-f BAMPE -p 0.05 -g hs'. The genomic annotation of ChIP-seq peaks were produced using the ChIPseeker v1.26.0 R package. BAM files were converted to bigWig files with CPM normalization using deepTools bamCoverage tool (version 3.3.5) and bigWig files were visualized in the WashU epigenome browser. Genomic position of OCT4 binding site on ITGA6 promoter was predicted on JASPAR database (https://jaspar.elixir.no/).

### Teratoma formation assay

To further verify the clearance effect of calcium- and magnesium-free (Ca-Mg-) BSS, a testicular teratoma formation model was selected for subsequent experiments. The BALB/c nude mice (6 weeks old, male) applied in this study were purchased from Vital River Laboratory Animal Technology Co., Ltd (401, Beijing, China) and were maintained under specific pathogen-free (SPF) conditions at Beijing Huilin Zegu Biotechnology Co., Ltd (Beijing, China). All procedures involving animals were reviewed and approved by the Institutional Animal Care and Use Committee (IACUC) of Beijing Huilin Zegu Biotechnology Co., Ltd (Approval No. HLZG-DWLL-2023-1205-02).

The experiment was divided into three groups: Group 1: co-cultured iPSCs and iMSCs (1×10^6^ cells, n = 6); Group 2: co-cultured iPSCs and iMSCs, after removal of iPSCs using DPBS(Ca-Mg-) (1×10^6^ cells, n = 6); Group 3: iMSCs (1×10^6^, n = 6).

Co-culture conditions similar to those used in *in vitro* experiments were applied. Briefly, 7.5×10^5^ iPSCs were first seeded onto Matrigel-coated 6-well plates (TeSR™-E8™ + 10 µM Y27632), and after 4 hours, the TeSR™-E8™ medium was replaced to remove Y27632. After another 20 hours of culture, 7.5×10^5^ iMSCs were seeded onto the plates containing the iPSCs using MSCM, and the co-culture was continued for an additional 8 hours. In Group 2, the procedures for removing iPSCs using DPBS(Ca-Mg-) were also similar to those in the *in vitro* experiment. The co-cultured cells were first washed twice with DPBS(Ca-Mg-), followed by treatment with 2 mL DPBS(Ca-Mg-) at 37 °C for 30 min. The co-cultured cells were first washed twice with DPBS(Ca-Mg-), followed by treatment with 2 mL DPBS(Ca-Mg-) at 37 °C for 30 min. Residual iPSCs were further removed by gentle pipetting, and the cells were then washed three times with DPBS(Ca-Mg-). Before injection, cells from all groups were digested with TrypLE at 37 °C for 5 min, centrifuged, and then resuspended in serum-free DMEM and Matrigel (1:1).

Subsequently, 20 µL (Containing 1×10^6^ cells) of the cell suspension was directly injected into the left testis of the nude mice using a microinjection syringe. After injection, the mice were maintained under standard conditions and regularly monitored for tumor formation in the testes. After 4 weeks, the mice were sacrificed, and both testes were dissected. The size and weight of both testes, as well as the Hematoxylin-Eosin (HE) staining results, were used to assess tumor formation.

### HE staining

First, tissue samples were fixed in 4% PFA at 4 °C for 24-48 hours. After fixation, the samples were dehydrated in a graded ethanol series (70%, 80%, 90%, 95%, and 100%) followed by clearing in xylene. Subsequently, the samples were embedded in paraffin using a paraffin embedding machine. The paraffin blocks were sectioned into 5 µm thick slices using a rotary microtome. These sections were mounted onto slides and dried at 60 °C for 1 hour to ensure proper adhesion of the tissue to the slides.

The HE staining was performed using the Hematoxylin-Eosin (HE) Stain Kit (G1120, Solarbio LIFE SCIENCES, Beijing, China) following the manufacturer's instructions. Briefly, the paraffin sections were deparaffinized in xylene and rehydrated through a graded ethanol series (100%, 95%, 80%, and 70%) before being rinsed with distilled water. Next, the sections were stained in hematoxylin solution for 3-5 min to visualize the cell nuclei. After rinsing with tap water, the sections were quickly differentiated in acid alcohol and rinsed again with water. The sections were then stained with eosin for 1-2 min to stain the cytoplasm. Following staining, the sections were dehydrated through a graded ethanol series (70%, 80%, 95%, and 100%), cleared in xylene, and mounted with a permanent mounting medium under a coverslip. The stained sections were observed under a light microscope to examine tissue morphology.

### Statistics and reproducibility

For all experiments, statistical methods were not used to predetermine sample size. Sample sizes are directly indicated in the figure legends. Image data were excluded from analysis if poor staining quality precluded image acquisition or analysis. In the animal studies, nude mice were randomly assigned to different groups, but other experiments were not randomized. No blinding was used in the analysis of any experiments. All quantitative data are presented as mean ± standard error of the mean (SEM). Unless otherwise stated, all experiments were repeated at least three times. Data were statistically analyzed and visualized using GraphPad Prism (version 8.0, GraphPad Software, CA, USA), with t-tests, one-way ANOVA, or two-way ANOVA. All statistically analyzed p-values are presented in figures or **Table S3**. Illustrations were created using BioRender and PowerPoint (Microsoft Corp., Washington, USA).

## Results

### Characteristics of PSCs

The characteristics of the PSCs applied in this study (iPSCs-001-5, iPSCs-006-1, EPSCs-001-5, EPSCs-006-1) were assessed through qPCR, immunofluorescence, ALP staining, and EB formation. As illustrated in **Figure S1A**, the colonies of the four PSCs appeared round or nearly round, with bright halos at the edges and tightly packed cells within. Compared to the negative control cells, HUVECs, all four PSC lines showed high gene-level expression of pluripotency markers OCT4, SOX2, Nanog, Klf17, and Dppa3 **(Figure S1B)**. As shown in **Figure S1C**, the PSC colonies stained intensely with the ALP substrate, indicating high levels of ALP expression, a characteristic marker of undifferentiated PSCs. The staining was uniform across the colonies.

The results of Immunofluorescence staining demonstrated at the protein level that all four groups of PSCs highly express pluripotency markers, including OCT4, SOX2, Nanog, TRA-1-60, and SSEA-4 (**Figure S1D**). To label PSCs for subsequent co-culture experiments, iPSCs were transduced with lentivirus carrying EGFP (**Figure S1E**). The transduced iPSCs expressed EGFP while maintaining the expression of pluripotent stem cell markers OCT4 and SOX2 (**Figure S1F**).

EB formation is a critical characteristic of PSCs and serves as a key method for assessing their pluripotency and differentiation potential. In this study, we evaluated the *in vitro* EB formation capacity of the primary PSC line used, iPSCs-001-5. As shown in **Figure S1G**, The PSCs successfully formed EBs when cultured under suspension conditions. From the first day of induction to the thirteenth day, the EBs were characteristically spherical with well-defined borders and gradually increasing in diameter. After culturing EBs on an adherent surface, the expression of markers for the three germ layers as well as pluripotency markers was assessed. Immunofluorescence staining confirmed the presence of ectodermal markers (PAX6, NESTIN and βIII-tubulin), mesodermal markers (PDGFα and α-smooth muscle actin), and endodermal marker (GATA4) within the EBs, indicating successful tri-lineage differentiation **(Figure S1H)**. The qPCR analysis further supported these findings, showing significant upregulation of lineage-specific genes such as DLX3 (ectoderm), PAX6 (ectoderm), Brachyury (mesoderm), and SOX17 (endoderm) compared to undifferentiated PSCs **(Figure S1I.)**. Additionally, a significant downregulation of pluripotency markers OCT4 and SOX2 was observed in the EBs compared to undifferentiated iPSCs **(Figure S1I)**.

### BSS (Ca-Mg-) is able to induce detachment of iPSCs *in vitro*, but not iPSC-derived differentiated cells (iDCs)

**Figure 1:**
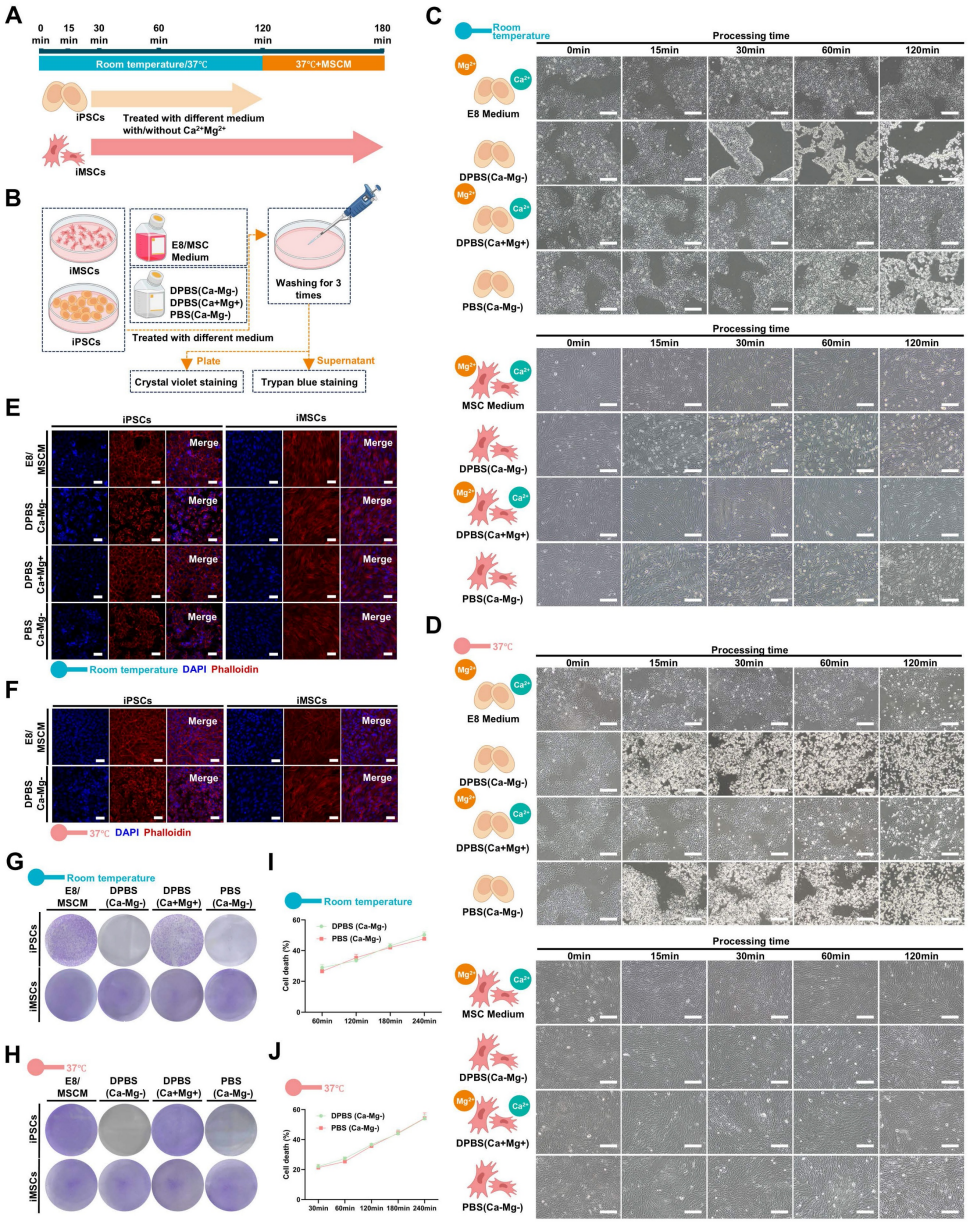
** BSS (Ca-Mg-) treatment rapidly induces detachment of iPSCs but does not affect iMSCs.** A-B. Schematic representation of the treatment protocol and evaluation criteria for iPSCs and iMSCs following BSS (Ca-Mg-) exposure; C-D. Light microscopy images showing the morphology of iPSCs and iMSCs after treatment with BSS (Ca-Mg-) at room temperature and 37 °C. Scale bars, 200 µm; E-F. Phalloidin staining illustrating the cytoskeletal structure of iPSCs and iMSCs after BSS (Ca-Mg-) treatment at room temperature (30 min) and 37 °C (15 min). Scale bars, 50 μm; G-H. Crystal violet staining indicates the presence of residual iPSCs and iMSCs after BSS (Ca-Mg-) treatment at room temperature and 37 °C; I-J. Trypan blue staining reveals the death rate of detached cells following BSS (Ca-Mg-) treatment at room temperature and 37 °C. Data for each point are presented as the mean ± SEM from three independent experiments (n = 3), and no statistical analysis was performed to assess differences between groups.

This study serendipitously discovered that a short exposure to BSS (Ca-Mg-) could cause PSCs to lose cell adhesion, similar to the effect of various enzymes or appropriate concentrations of EDTA used for cell dissociation. Interestingly, this phenomenon was not observed in iMSCs. In summary, under the same conditions, iMSCs were found to resist the detachment-inhibiting effects of BSS (Ca-Mg-). The iPSCs-001-5 and iMSCs were selected as representative models for PSCs and PSC-derived differentiated cells, respectively, to explore the conditions for BSS (Ca-Mg-) treatment. Initially, iPSCs and iMSCs were treated with DPBS(Ca-Mg-), PBS(Ca-Mg-), DPBS(Ca+Mg+), and their respective maintenance media (E8 or MSCM) at 37 °C or room temperature for 15 to 120 min. Cell adhesion was then assessed through light microscopy, Phalloidin staining and crystal violet staining **(Figure 1A-B)**.

At room temperature, treatment with DPBS(Ca-Mg-) and PBS (Ca-Mg-) for 60 min caused iPSCs to lose adhesion, leading to cell shrinkage and a weakening of cell-cell connections **(Figure 1C.)**. As the treatment time increased, some cells detached from the culture dish and became suspended in the two BSS (Ca-Mg-) solutions **(Figure 1C)**. In contrast, iPSCs treated with DPBS (Ca+Mg+) and E8 showed no significant changes in colony morphology or adhesion compared to before treatment. Notably, within 120 min of treatment, iMSCs did not undergo extensive cell detachment, regardless of whether calcium and magnesium ions were present **(Figure 1C)**. To further explore the optimal conditions for BSS (Ca-Mg-) treatment, iPSCs and iMSCs were treated under the same conditions at 37 °C as at room temperature. The results showed that at 37 °C, BSS (Ca-Mg-) required only 30 min to induce iPSC detachment (compared to 60 min at room temperature) **(Figure 1D)**. However, within 120 min of treatment at 37 °C, the presence or absence of calcium and magnesium ions did not negatively impact iPSC adhesion or iMSC adhesion **(Figure 1D).**

Phalloidin staining was utilized to visualize the organization of F-actin within the cells. This study further investigated the impact of BSS (Ca-Mg-) on the cytoskeleton under adherent conditions using Phalloidin staining. The results showed that iPSCs and iMSCs treated with E8/MSCM and/or DPBS (Ca+Mg+) at room temperature and 37 °C maintained well-organized cytoskeletal structures **(Figure 1E-F)**. In contrast, iPSCs treated with DPBS(Ca-Mg-) and/or PBS (Ca-Mg-) exhibited significant cytoskeletal contraction, while iMSCs showed no notable changes **(Figure 1E-F)**. This finding supports the observation under light microscopy that BSS (Ca-Mg-) selectively induces detachment in iPSCs.

After treatment at room temperature (60 min) and 37 °C (30 min), cells in each group were gently pipetted and washed, followed by assessment of cell retention through crystal violet staining. As shown in **Figure 1G-H,** BSS (Ca-Mg-) treatment effectively cleared iPSCs at both room temperature and 37 °C without affecting the adhesion of iMSCs. Trypan blue staining was performed on cells treated with DPBS (Ca-Mg-) and PBS (Ca-Mg-) at room temperature and 37 °C to preliminarily explore the cause of cell detachment. The results indicated that treatment with DPBS(Ca-Mg-) and PBS (Ca-Mg-) at 37 °C for 30 min led to the death of approximately 20%-25% of iPSCs, while treatment at room temperature for 60 min caused 25%-30% iPSC death. Therefore, the detachment of iPSCs induced by BSS (Ca-Mg-) treatment might be partially due to the rapid induction of cell death, although other mechanisms may also be involved.

In addition to iPSCs-001-5, this study further investigated whether the response of PSCs to BSS (Ca-Mg-) was consistent across three additional PSC cell lines (iPSCs-006-1, EPSC-001-5, and EPSCs-006-1). The results indicated that both DPBS(Ca-Mg-) and PBS (Ca-Mg-) effectively caused all three types of PSCs to lose adhesion under 37 °C or room temperature conditions (37 °C for 30 min or room temperature for 60 min) **(Figure S2A-F)**. This was further confirmed by subsequent crystal violet staining **(Figure S2G-I)**. The condition of treating cells with DPBS(Ca-Mg-) at 37 °C for 30 min was chosen for subsequent experiments for the following reasons: 1) DPBS(Ca-Mg-) and PBS (Ca-Mg-) had similar effects; 2) To achieve the same clearance effect, the 37 °C condition required a shorter time (30 min); 3) 37 °C is the normal culture temperature for cells.

Using the established experimental conditions, this study examined other iDCs to determine whether their resistance to DPBS(Ca-Mg-) was consistent with that of iMSCs. First, the iPSCs were differentiated into iFCs, iOBs, and iEPCs using well-established methods **(Figure S3A)**. Treatment with DPBS(Ca-Mg-) at 37 °C for 30 min did not cause significant changes in iFCs, iOBs, or iEPCs **(Figure S3B)**. This suggests that DPBS(Ca-Mg-) may be used for the removal of residual PSCs following the culture of at least four types of iDCs (iMSCs, iFCs, iOBs, and iEPCs).

### DPSC (Ca-Mg-) selectively removes PSCs in iPSCs/iDCs co-culture system *in vitro*

**Figure 2:**
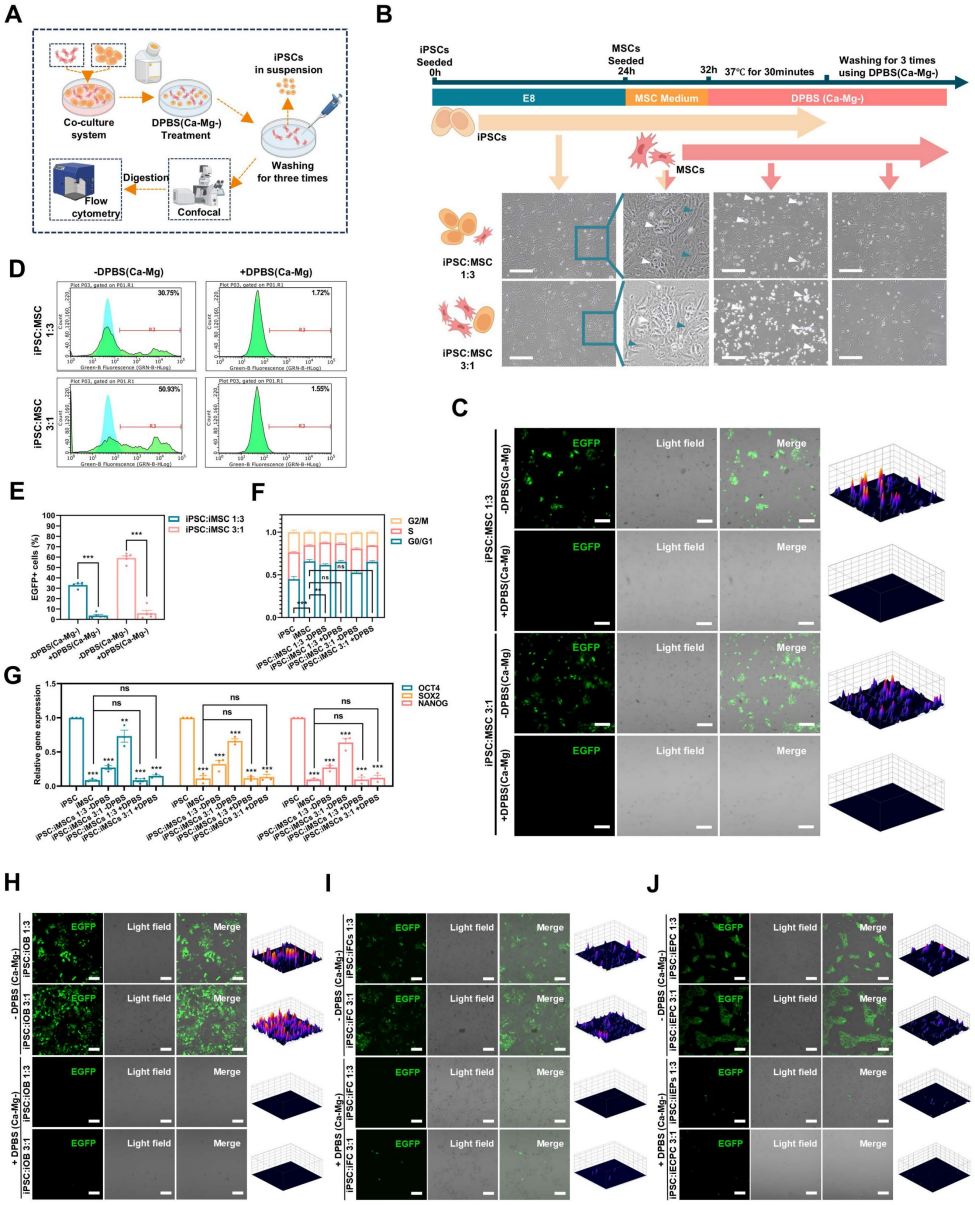
** DPBS (Ca-Mg-) efficiently and selectively removes iPSCs from iPSCs/iDCs co-culture systems.** A. Schematic illustration of the co-culture system setup, DPBS (Ca-Mg-) treatment, and evaluation criteria; B. Light microscopy images of the iPSCs/iMSCs co-culture (white arrows: iPSCs; blue arrows: iMSCs). Scale bars, 200 µm; C. Fluorescence images of the iPSCs/iMSCs co-culture before and after DPBS (Ca-Mg-) treatment (iPSCs express EGFP, iMSCs do not). Scale bars, 100 µm; D-E. Flow cytometry analysis of adherent cells before and after DPBS (Ca-Mg-) treatment (iPSCs express EGFP, iMSCs do not). The data are presented as the mean ± SEM from five biological replicates (n = 5). Statistical significance was determined using two-way ANOVA, with *p < 0.05; **p < 0.01; ***p < 0.001; F. Cell cycle analysis of adherent cells before and after DPBS (Ca-Mg-) treatment; The results from three independent experiments (n = 3) are presented as mean ± SEM. One-way ANOVA was used for comparisons between groups. No significance (ns) p > 0.05; *p < 0.05; **p < 0.01; ***p < 0.001; G. qPCR analysis of adherent cells, iPSCs, and iMSCs before and after DPBS (Ca-Mg-) treatment. The data from three independent experiments (n = 3) are expressed as mean ± SEM. Group comparisons were performed using one-way ANOVA. Statistical significance is indicated as follows: no significance (ns) p > 0.05, *p < 0.05, **p < 0.01, ***p < 0.001; H-J. Fluorescence images of iPSCs/iOBs, iPSCs/iFCs, and iPSCs/iEPCs co-culture systems before and after DPBS (Ca-Mg-) treatment (iPSCs express EGFP, while iOBs, iFCs, and iEPCs do not). Scale bars, 100 µm.

To simulate the residual PSCs in a realistic induction environment, a co-culture model of iPSCs and iDCs was established **(Figure 2A; Figure S3A)**. Briefly, EGFP-transduced iPSCs were initially seeded in Matrigel-coated 6-well plates using E8 medium with 10 μM Y27632. After 4 hours, the medium was changed to E8 without Y27632, and the cells were cultured for an additional 20 hours. Then, different iDCs were seeded onto the iPSC-adherent plates using their respective differentiation media and co-cultured for 8 hours to establish the iPSC-iDC co-culture system **(Figure 2b; Figure S3C)**. The seeding ratio of iPSCs to iDCs was either 1:3 (3×10^5^:9×10^5^) or 3:1 (9×10^5^:3×10^5^).

As shown in **Figure 2B,** a co-culture system of iPSCs and iMSCs was successfully established, where iPSCs exhibited clonal-like growth, surrounded by scattered spindle-shaped iMSCs (blue arrows: iMSCs; white arrows: iPSCs). After treatment with DPBS(Ca-Mg-), some cells lost adhesion and detached, floating in the DPBS(Ca-Mg-) solution. Following the PBS washing, only spindle-shaped cells remained adherent at the bottom of the dish. Confocal microscopy revealed that the majority of EGFP-positive cells (iPSCs) were effectively cleared **(Figure 2C)**. This observation was further confirmed by subsequent flow cytometry analysis, which showed that the percentage of EGFP-positive cells in the co-cultures with iMSCs at different ratios (1:3 and 3:1) significantly decreased from 32.3% and 58.15% to 4.12% and 7.44%, respectively **(Figure 2D-E)**.

The **Figure 2F** illustrated the differences in the cell cycle distribution among the iPSCs group, iMSCs group, the two co-culture groups, and the two co-culture groups treated with DPBS(Ca-Mg-). The results indicated that the proportion of cells in the G0/G1 phase was significantly lower in the iPSCs group compared to the iMSCs group, while the G0/G1 phase proportion in the co-culture groups was intermediate between that of iPSCs and iMSCs **(Figure 2F)**. After DPBS(Ca-Mg-) treatment, the G0/G1 phase proportion in the co-culture groups increased and showed no statistical difference compared to the iMSCs group** (Figure 2F)**. Similar trends were observed in the expression of pluripotency genes (OCT4, SOX2, and Nanog) across the same groups, as detected by qPCR. Specifically, the expression levels of all three pluripotency genes were significantly higher in iPSCs compared to iMSCs, while the co-culture groups showed intermediate expression levels between iPSCs and iMSCs **(Figure 2G)**. Following DPBS(Ca-Mg-) treatment, the expression of pluripotency genes in the co-culture groups decreased to levels comparable to those in the iMSCs group **(Figure 2G)**.

The cell cycle analysis and qPCR results suggested that the vast majority of cell population with a lower G0/G1 phase proportion and higher pluripotency gene expression was successfully removed by DPBS(Ca-Mg-). This is consistent with the fluorescence microscopy and flow cytometry findings, where the proportion of EGFP-positive iPSCs was markedly reduced. Thus, in the co-culture system of iPSCs and iMSCs, DPBS(Ca-Mg-) was demonstrated to selectively remove iPSCs.

In addition to co-culturing iMSCs, this study also established co-culture systems of EGFP-positive iPSCs with iOBs, iFCs, and iEPCs **(Figure S3C)**. Similar to the iPSCs and iMSCs co-culture, iPSCs exhibited clonal-like growth, surrounded by differentiated cells (blue arrows: iDCs, including iOBs, iFCs, and iEPCs; white arrows: iPSCs)** (Figure S3C)**. After treatment with DPBS(Ca-Mg-), a portion of the cells were dissociated **(Figure S3C)**. Following PBS washing, fluorescence analysis of the remaining cells in the dish was conducted. As shown in **Figure 2H-J**, after DPBS(Ca-Mg-) treatment, the majority of EGFP-positive (iPSCs) cells in each co-culture group were cleared. Flow cytometry provided further evidence supporting the selective clearance of iPSCs by DPBS(Ca-Mg-) in co-cultures with iDCs. Specifically, in the co-culture groups of iPSCs and iOBs, the percentage of EGFP-positive cells decreased from 16.02% and 38.34% to 2.25% and 2.36%, respectively **(Figure S3D)**. In the iPSCs and iFCs co-culture groups, the EGFP-positive rate dropped from 23.14% and 50.33% to 4.83% and 10.33% **(Figure S3D)**. Lastly, in the iPSCs and iEPCs co-culture groups, the EGFP-positive rate decreased from 24.88% and 52.26% to 5.26% and 7.78%, respectively **(Figure S3D)**.

In this section, the study simulates PSCs residuals under real differentiation conditions *in vitro* through a co-culture system. In the co-culture systems of iPSCs with four different iDCs (iMSCs, iOBs, iFCs, and iEPCs), DPBS(Ca-Mg-) effectively and selectively cleared the iPSCs.

### The iPSCs/iMSCs co-culture system treated with DPBS(Ca-Mg-) does not induce teratoma formation *in vivo*

**Figure 3:**
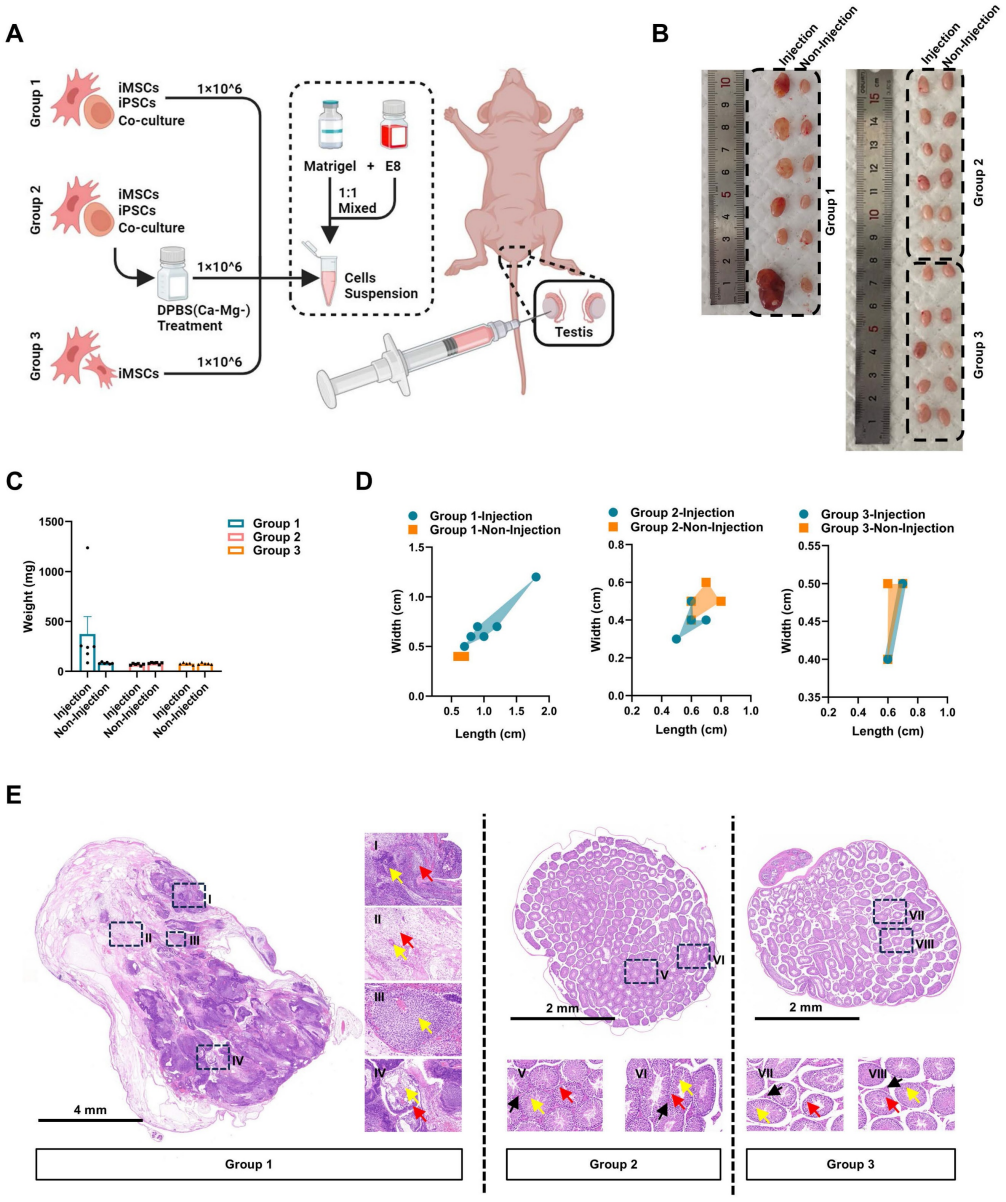
** DPBS (Ca-Mg-) treated iPSCs/iMSCs co-culture system does not induce teratoma formation *in vivo*.** A. Schematic and grouping of the *in vivo* teratoma formation experiment. B. Images of nude mice testes after injection with different cell groups (left testis: injected with cells; right testis: no injection; Group 1, n = 6; Group 2, n = 5; Group 3, n = 6). C. Testis weights of nude mice injected with different cell groups. Data are presented as mean ± SEM, but no statistical analysis was performed (Group 1, n = 6; Group 2, n = 5; Group 3, n = 6). DPBS (Ca-Mg-) treated iPSCs/iMSCs co-culture system does not induce teratoma formation *in vivo*. D. Length and width of teratomas formed in nude mice injected with different cell groups (Group 1, n = 6; Group 2, n = 5; Group 3, n = 6). E. HE staining results of the testis injected with iPSCs/iMSCs (left side) and DPBS (Ca-Mg-) treated iPSCs/iMSCs (left side), along with the right-side testis that did not receive cell injections. I. Immature neural tube (yellow arrow, ectoderm), immature nerve tissue (red arrow, ectoderm); II. Loose fibrous connective tissue (red arrow, mesoderm), blood vessels (yellow arrow, mesoderm); III. Immature cartilage tissue (yellow arrow, mesoderm); IV. Immature differentiated squamous epithelium (yellow arrow, ectoderm), bronchial mucosal epithelium (red arrow, endoderm); V-VIII. Spermatogenic cells (yellow arrow), Leydig cells (red arrow), sperm (black arrow).

Further evaluation of the selective clearance ability of DPBS(Ca-Mg-) on iPSCs was conducted using an *in vivo* teratoma formation assay. In this study, co-cultured iPSCs/iMSCs, DPBS(Ca-Mg-)-treated co-cultured iPSCs/iMSCs, and iMSCs alone were injected into the left testicular region of nude mice **(Figure 3A)**. Each group consisted of 6 nude mice. However, one nude mice in Group 2 died due to an anesthesia-related incident. Four weeks post-injection, both the left (injection) and right (non-injection) testicles were harvested for analysis **(Figure 3B)**. No significant differences in weight were observed in the right testicles across all groups **(Figure 3C)**.

In five out of six mice injected with the co-cultured iPSCs/iMSCs, the left testicle showed an increase in weight and dimensions (length and width) compared to the right testicle **(Figure 3B-D)**. The weights of the enlarged left testicles ranged from 176.6 mg to 1239.8 mg, while the right testicles weighed between 77.5 mg and 96.4 mg **(Figure 3C)**. Histological sections of the enlarged left testicles confirmed the presence of tissues derived from all three germ layers: ectoderm, mesoderm, and endoderm. Ectodermal derivatives included immature neural tube, neural tissue, and immature differentiated squamous epithelium. Mesodermal tissues comprised loose fibrous connective tissue, cartilage, and vascular. Endodermal structures clearly exhibited bronchial mucosal epithelium. However, the overall structure of the right testis in each group and the left testis in Group 2 was basically normal, the structure of the seminiferous tubules was clear, and the spermatogenic cells in the seminiferous tubules were arranged regularly. In contrast, no significant increase in weight or dimensions was observed in the left testicles of mice injected with DPBS(Ca-Mg-)-treated co-cultured iPSCs/iMSCs or iMSCs alone compared to their right testicles **(Figure 3B-D)**.

### Characterization of iMSCs is not affected by DPBS(Ca-Mg-) treatment

The above experiments provide both *in vitro* and *in vivo* evidence for the selective clearance of iPSCs by DPBS(Ca-Mg-). Subsequently, this study used iMSCs as a model to preliminarily explore the impact of DPBS(Ca-Mg-) treatment on the characteristics of iDCs **(Figure S4A)**. Similar to adult MSCs, iMSCs also exhibit tri-lineage differentiation potential and self-renewal capabilities, while expressing MSC markers 39. As shown in **Figure S4B**, regardless of whether iMSCs were treated with DPBS(Ca-Mg-) at 37 °C or room temperature, when the iMSCs were subsequently cultured in MSCM at 37 °C, they maintained their typical spindle-shaped morphology without any noticeable cell death.

After DPBS(Ca-Mg-) treatment, iMSCs continued to express high levels of MSC markers, including CD44 (99.87%), CD73 (96.67%), CD90 (99.92%), CD105 (99.40%), and CD166 (99.88%), while remaining negative for MSC-negative markers such as CD34 (0.08%), CD45 (0.08%), and HLA-DR (0.11%). This marker expression profile is consistent with that observed before DPBS(Ca-Mg-) treatment **(Figure S4C)**. On the other hand, DPBS(Ca-Mg-) treatment did not affect the tri-lineage differentiation potential of iMSCs. Alizarin Red staining (for osteogenic differentiation), ALP staining (for osteogenesis), Oil Red O staining (for adipogenesis), and Alcian Blue staining (for chondrogenesis) of both untreated and DPBS(Ca-Mg-)-treated iMSCs after lineage-specific induction support this result **(Figure S4D-G)**.

Additionally, this study compared the migration and self-renewal abilities of iMSCs before and after DPBS(Ca-Mg-) treatment. The wound healing assay results showed no significant differences in the migratory capacity between DPBS(Ca-Mg-)-treated iMSCs and untreated iMSCs **(Figure S4H-I)**. In both the colony formation assay and CCK8 assay, DPBS(Ca-Mg-)-treated iMSCs maintained normal colony formation and proliferation abilities, consistent with those of untreated iMSCs **(Figure S4J-L)**. These findings suggest that short-term DPBS(Ca-Mg-) treatment does not affect iMSC characteristics, indicating that the application of DPBS(Ca-Mg-) to remove residual PSCs after iMSC induction does not hinder subsequent application of iMSCs. However, this study did not further investigate the effects of DPBS(Ca-Mg-) treatment on other iDCs, which requires future research.

### RNA-seq revealed differences in adhesion signaling and associated integrin signaling between iPSCs and iMSCs

**Figure 4:**
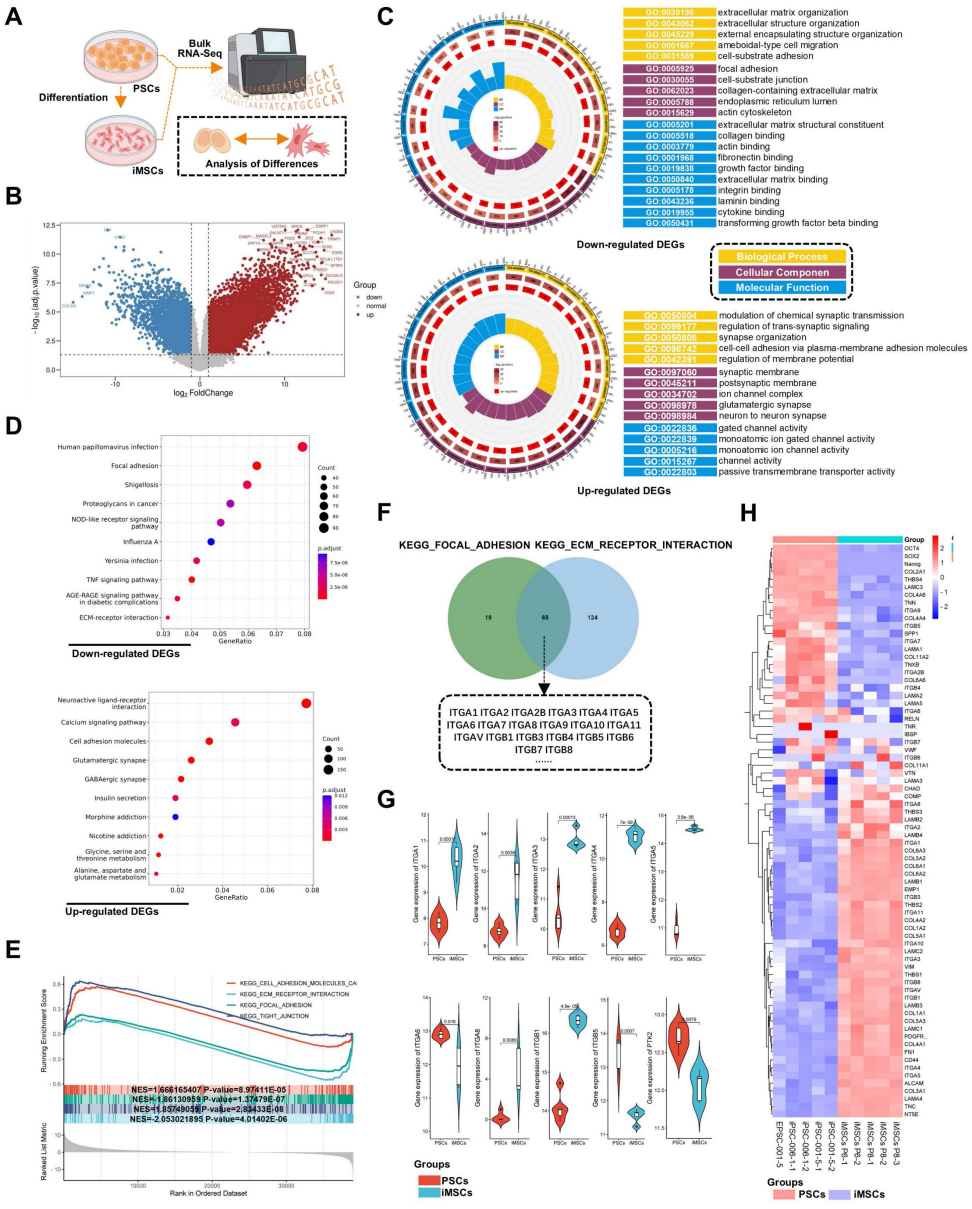
** RNA-seq analysis of PSCs and iMSCs reveals differential gene expression.** A. Schematic diagram showing the workflow for RNA-seq; B. Volcano plot displaying the distribution of differentially expressed genes (DEGs). Genes with a log2|FC| > 1 and adjusted p-value < 0.05 are considered significantly differentially expressed (upregulated genes are marked in red, downregulated genes in blue); C. Gene Ontology (GO) enrichment analysis for upregulated and downregulated DEGs, showing significantly enriched biological processes; D. KEGG pathway enrichment scatter plot. The X-axis represents the GeneRatio, and the Y-axis shows the KEGG pathways. The size of the points corresponds to the number of DEGs enriched in each pathway, and the color gradient (red to blue) represents the statistical significance (adjusted p-value), with redder colors indicating higher significance; E. Gene Set Enrichment Analysis (GSEA) of RNA-seq data, identifying enriched biological pathways. The X-axis represents the ranking of genes between iPSCs and iMSCs, while the Y-axis indicates the Enrichment Score (ES). The peak of the curve indicates where the gene set is most enriched, with higher ES signifying stronger enrichment; F. Venn diagram showing the overlap of genes enriched in two GSEA-identified pathways (KEGG_FOCAL_ADHESION and KEGG_ECM_RECEPTOR_INTERACTION). These genes play crucial roles in both pathways; G. Violin plot comparing the expression levels of common integrins in PSCs and iMSCs. The p-values from the Wilcoxon test are shown in the figure; H. Heatmap displaying the expression of pluripotency genes, MSC marker genes, and adhesion-related genes between PSCs and iMSCs.

To explore the mechanisms underlying the differential response of PSCs and four types of iDCs to DPBS (Ca-Mg-), this study first compared the transcriptomic profiles of iMSCs and PSCs via RNA-seq **(Figure 4A)**. Differentially expressed genes (DEGs) between iMSCs and PSCs were identified using criteria of adjusted p-value < 0.05 and |log2 fold change| > 1 **(Figure 4B)**. GO enrichment analysis was performed separately for the upregulated and downregulated DEGs. Among the upregulated DEGs, many GO terms related to cell adhesion were enriched, including integrin binding, collagen binding, actin binding, fibronectin binding, laminin binding, and focal adhesion **(Figure 4C)**. KEGG pathway analysis further revealed that the upregulated DEGs in iMSCs were significantly enriched in pathways such as focal adhesion, TNF signaling pathway, and ECM-receptor interaction, all of which are also related to cell adhesion **(Figure 4D)**. Both GO enrichment analysis and KEGG pathway analysis suggest potential differences in adhesion capability between iMSCs and iPSCs.

GSEA (Gene Set Enrichment Analysis) was used to further investigate the biological pathways and processes involved in iPSCs and iMSCs. Interestingly, iMSCs showed enrichment in pathways related to cell adhesion with the extracellular matrix, such as KEGG_FOCAL_ADHESION and KEGG_ECM_RECEPTOR_INTERACTION **(Figure 4E)**. In contrast, iPSCs were enriched in pathways associated with cell-cell adhesion, such as KEGG_TIGHT_JUNCTION **(Figure 4E)**. This suggested that iMSCs may have stronger adhesion to the extracellular matrix, while iPSCs are more inclined towards cell-cell adhesion. To investigate the genes in iMSCs that play crucial roles in cell-extracellular matrix adhesion, the genes enriched in the two pathways (KEGG_FOCAL_ADHESION and KEGG_ECM_RECEPTOR_INTERACTION) were intersected **(Figure 4F)**. The results revealed that several integrin genes, such as ITGA6 and ITGB1, play significant roles in both pathways **(Figure 4F)**. The heatmap in **Figure 4H** illustrated the gene expression profiles of certain genes in PSCs and iMSCs, including pluripotency genes (such as OCT4, SOX2, and Nanog), MSCs markers (like CD44, NT5E, and ALCAM), and genes associated with cell adhesion.

Several integrins, including ITGA6, ITGB1, ITGA3, ITGA5, and ITGA1, are highly expressed in pluripotent stem cells 43. Consistent with their findings, ITGA6 was the only integrin among these that exhibited significantly higher expression in PSCs compared to iMSCs in this study **(Figure 4G)**. The expression patterns of other integrins in both PSCs and iMSCs are presented in **Figure 4G** and **Figure S5A**. In summary, through bioinformatics analysis, this study highlighted alterations in pluripotency genes and integrin signaling, particularly ITGA6.

**Figure 5:**
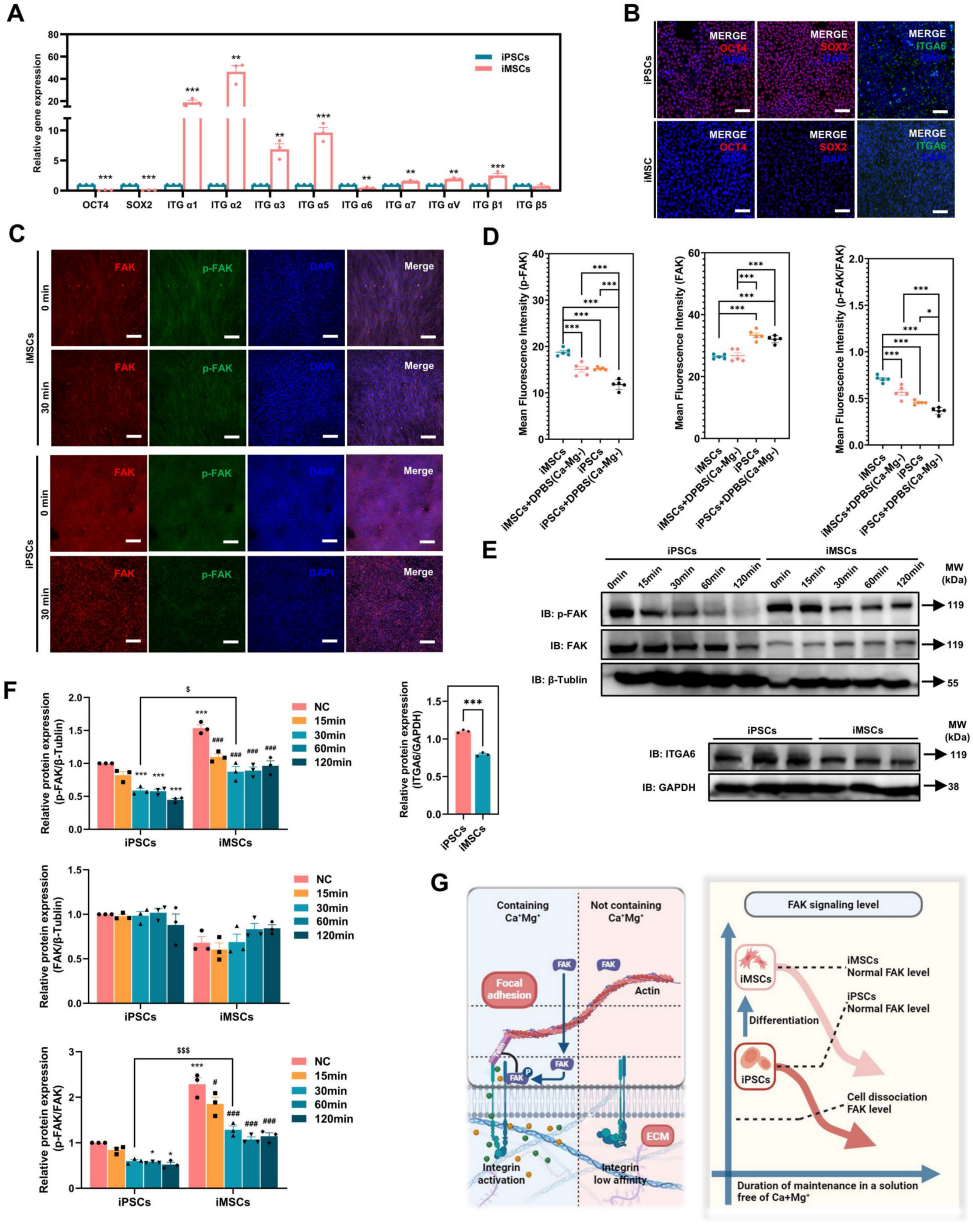
** DPBS (Ca-Mg-) treatment rapidly modulates FAK signaling in iPSCs and iMSCs.** A. qPCR analysis of the expression levels of common pluripotency and integrin genes in iPSCs and iMSCs. Data are presented as mean ± SEM from three independent experiments (n = 3), with statistical significance determined using Student's t-test (*p < 0.05; **p < 0.01; ***p < 0.001); B. Immunofluorescence images showing the expression of OCT4, SOX2, and ITGA6 in iPSCs and iMSCs. Scale bars, 100 µm; C-D. Immunofluorescence images and quantitative analysis of p-FAK and FAK in iPSCs and iMSCs before and after DPBS (Ca-Mg-) treatment. Quantification is based on five independent experiments (n = 5), with data presented as mean ± SEM and analyzed using one-way ANOVA, *p < 0.05; **p < 0.01; ***p < 0.001. Scale bars, 200 µm; E. Western blot showing the expression levels of p-FAK, FAK, and ITGA6 before and after DPBS (Ca-Mg-) treatment; F. Quantification of p-FAK, FAK, and ITGA6 expression. Statistical analysis for p-FAK, FAK, and p-FAK/FAK ratios was performed using two-way ANOVA (n = 3), while ITGA6 expression was analyzed using Student's t-test (n = 3). Data are presented as mean ± SEM, * compared with iPSCs, # compared with iMSCs, $ compared with iPSCs: 30min (*/#/$ p < 0.05; **/##/$$ p < 0.01; ***/###/$$$ p < 0.001); G. Schematic diagram illustrating the modulation of FAK signaling by DPBS (Ca-Mg-) treatment.

Notably, integrin signaling was able to mediate cell adhesion by recruiting and activating signaling proteins, transmitting both mechanical and chemical signals into the cell's interior 44. FAK, a cytoplasmic tyrosine kinase, played a crucial role as a downstream component in this process 45. Previous studies had reported the regulation of ITGA6 expression by the pluripotency gene OCT4 46. Therefore, this study hypothesized that the differences in OCT4, ITGA6, and FAK signaling between iPSCs and iMSCs led to differences in cell adhesion and their response to DPBS (Ca-Mg-). However, the upstream and downstream regulatory mechanisms of OCT4, ITGA6, and FAK signaling remained unclear. The expression profiles of the pluripotency gene SOX2 and other common integrin genes are presented in **Figure 5A**.

### Differences in OCT4 and ITGA6 expression and FAK signaling between PSCs and iDCs

Consistent with the RNA-seq results, both qPCR and immunofluorescence confirmed significant downregulation of OCT4 and ITGA6 in iMSCs at the gene and protein levels **(Figure 5A-B)**. The lower ITGA6 protein expression in iMSCs compared to iPSCs was further validated by WB analysis **(Figure 5E-F)**. Additionally, immunofluorescence and WB assays were used to assess FAK signaling activation in both iPSCs and iMSCs. Quantification of the immunofluorescence images revealed that iPSCs exhibited higher FAK levels, but p-FAK expression and the p-FAK/FAK ratio were significantly lower in iPSCs compared to iMSCs** (Figure 5C-D)**, which was consistent with the WB results **(Figure 5E-F)**. This indicates that FAK signaling is more actively engaged in iMSCs than in iPSCs. iMSCs possess mesodermal phenotypes and potential for differentiation into various mesodermal lineages **(Figure S4)**. Whether the differences in OCT4, ITGA6, and FAK signaling between iPSCs and iMSCs also occur during differentiation into other germ layers (beyond mesoderm) was further explored in subsequent experiments.

The study induced the directed differentiation of iPSCs into neuroectoderm and mesendoderm lineages following previously reported protocols **(Figure S6A).** The differentiated cells were characterized via qPCR and immunofluorescence. The qPCR results showed that neuroectoderm cells exhibited higher expression levels of the neuroectoderm marker PAX6 compared to iPSCs **(Figure S6B)**. In mesendoderm cells, both Brachyury (mesoderm marker) and SOX17 (endoderm marker) were significantly upregulated **(Figure S6B)**. Immunofluorescence further confirmed the successful induction of neuroectoderm and mesendoderm cells, with PAX6 and NESTIN (neuroectoderm markers) being positive in neuroectoderm cells, and GATA4 (endoderm marker) positive in mesendoderm cells **(Figure S6D)**.

As shown in **Figure S6B**, OCT4 expression was significantly reduced in both differentiated lineages compared to iPSCs** (Figure S6B-C)**. Additionally, qPCR, immunofluorescence, and WB results demonstrated that differentiation into both germ layers led to decreased ITGA6 expression **(Figure S6B-C, G-H)**. The activation of FAK signaling in these differentiated cells was also examined. Both immunofluorescence and WB data showed that p-FAK levels and the p-FAK/FAK ratio were significantly higher in the neuroectoderm and mesendoderm cells than in iPSCs **(Figure S6E-H)**. In summary, these findings suggest that differentiation of iPSCs (into mesoderm, mesendoderm, or neuroectoderm) leads to reduced expression of OCT4 and ITGA6 and increased activation of FAK signaling.

### Differences in FAK signaling led to variations in the resistance of iPSCs and iMSCs to BBS(Ca-Mg-)

In the above section, it was revealed that the FAK signaling pathway is more strongly activated in iMSCs compared to iPSCs under normal culture conditions. Building on this, the study further investigated the rapid regulation of the FAK signaling in both iMSCs and iPSCs upon DPBS (Ca-Mg-) treatment, to explain why this treatment causes rapid detachment in iPSCs but not in iMSCs. **Figure 5C-D** show the immunofluorescence staining results of p-FAK and FAK in iMSCs and iPSCs after DPBS (Ca-Mg-) treatment (37 °C, 30 min). The results indicate that after DPBS (Ca-Mg-) treatment, both p-FAK levels and the p-FAK/FAK ratio were significantly downregulated in iPSCs and iMSCs **(Figure 5C-D)**. However, it is noteworthy that the p-FAK levels and the p-FAK/FAK ratio remained significantly higher in DPBS (Ca-Mg-)-treated iMSCs compared to treated iPSCs **(Figure 5C-D)**.

Subsequently, the study examined FAK signaling levels over different time points (15 to 120 min) after DPBS (Ca-Mg-) treatment at 37°C via WB. As shown in **Figure 5E-F**, p-FAK levels and the p-FAK/FAK ratio in iPSCs gradually decreased as the DPBS (Ca-Mg-) treatment duration increased. In contrast, p-FAK levels and the p-FAK/FAK ratio in iMSCs decreased during the first 30 min of treatment, but then stabilized at relatively high levels over the next 90 min **(Figure 5E-F)**. Consistent with the immunofluorescence results, p-FAK and p-FAK/FAK in iMSCs after 30 min of DPBS (Ca-Mg-) treatment was significantly higher than in iPSCs treated for the same duration** (Figure 5E-F)**. Based on these findings, the study hypothesized that the differences in FAK signaling directly contribute to the varied responses of iPSCs and iMSCs to DPBS (Ca-Mg-) treatment. These differences in FAK signaling between the two cell types may stem from two key factors: (1) a higher level of FAK activation in iMSCs under normal culture conditions, and (2) the early downregulation of FAK signaling in iMSCs during the initial phase of DPBS (Ca-Mg-) treatment, followed by sustained stability thereafter **(Figure 5G)**.

**Figure 6:**
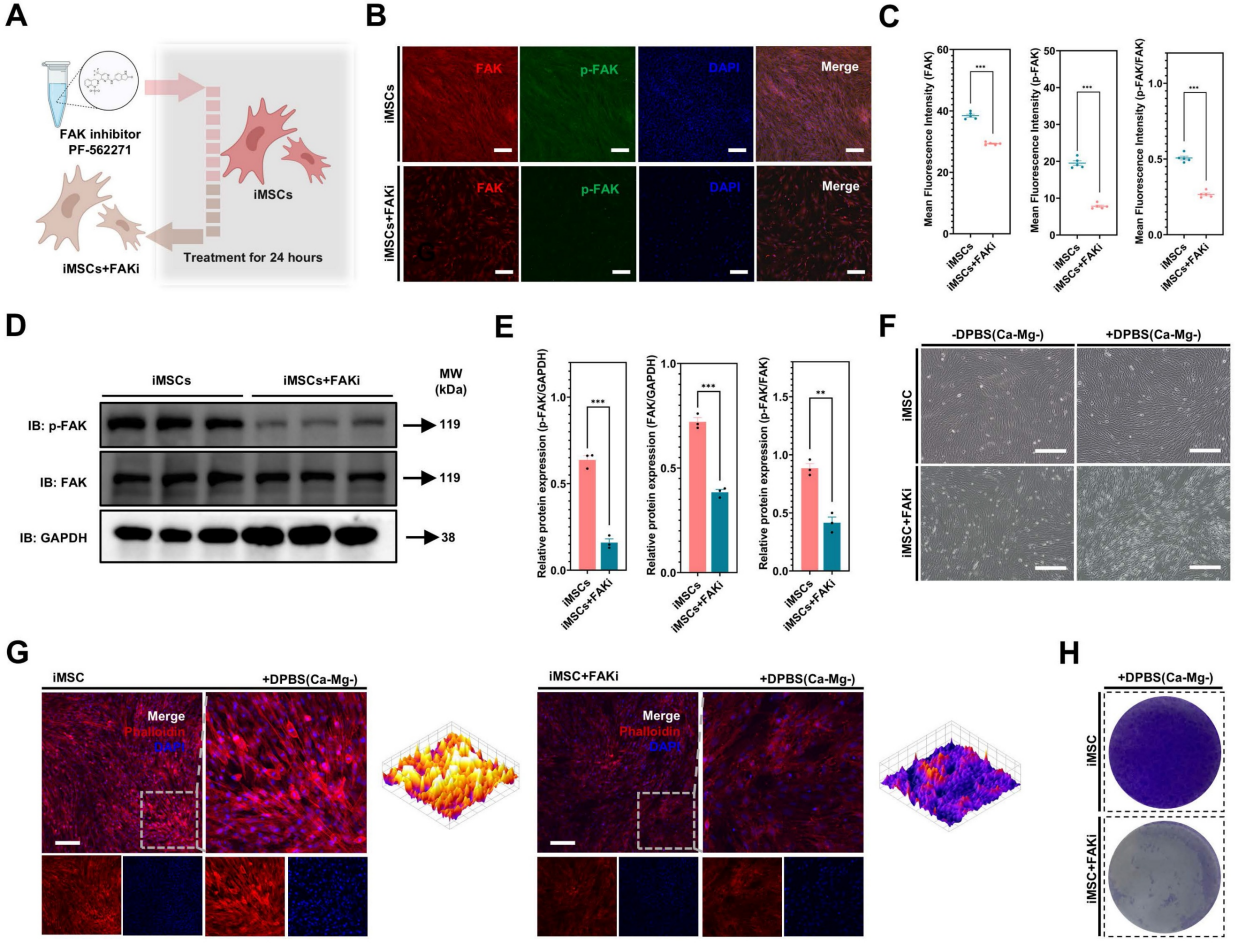
** Inhibition of FAK signaling in iMSCs weakens their resistance to DPBS (Ca-Mg-) treatment.** A. Schematic diagram illustrating the strategy for inhibiting FAK signaling in iMSCs using the PF-562271; B-C. Immunofluorescence images and quantitative analysis of p-FAK and FAK expression in iMSCs before and after FAKi treatment (n = 5). Data are presented as mean ± SEM, with statistical comparisons between groups performed using Student's t-test (*p < 0.05; **p < 0.01; ***p < 0.001). Scale bars, 100 µm; D-E. Western blot analysis and quantification of p-FAK and FAK expression in iMSCs before and after FAKi treatment (n = 3). Data are presented as mean ± SEM, with statistical comparisons performed using Student's t-test (*p < 0.05; **p < 0.01; ***p < 0.001); F. Bright-field images showing the morphology of iMSCs and FAKi-treated iMSCs before and after DPBS (Ca-Mg-) treatment. Scale bars, 200 µm; G. Phalloidin fluorescence images showing the cytoskeleton structure of iMSCs and FAKi-treated iMSCs before and after DPBS (Ca-Mg-) treatment. Scale bars, 100 µm; H. Crystal violet staining images of iMSCs and FAKi-treated iMSCs before and after DPBS (Ca-Mg-) treatment.

To test this hypothesis, the study pretreated iMSCs with a FAK phosphorylation inhibitor (PF-562271, 1µM, FAKi) for 24 hours to reduce FAK signaling activation in iMSCs **(Figure 6A)**. PF-562271 is a potent, ATP-competitive and reversible FAK kinase inhibitor that inhibits FAK phosphorylation in a dose-dependent manner 47-49. Immunofluorescence and WB results confirmed that FAKi treatment significantly downregulated p-FAK levels, FAK levels, and the p-FAK/FAK ratio in iMSCs **(Figure 6B-E)**. After DPBS (Ca-Mg-) treatment, iMSCs pretreated with FAKi exhibited noticeable cell shrinkage **(Figure 6F)**, which was further validated by phalloidin staining. In DPBS (Ca-Mg-)-treated iMSCs, the cytoskeleton remained intact, tightly arranged, and uniformly distributed in the cytoplasm. However, in FAKi-pretreated iMSCs, DPBS (Ca-Mg-) treatment led to disorganized and disrupted cytoskeletal structures **(Figure 6G)**. Subsequent crystal violet staining confirmed that FAKi-pretreated iMSCs lost their resistance to DPBS (Ca-Mg-) treatment and were effectively cleared **(Figure 6H)**, similar to the response observed in iPSCs **(Figure 1H)**. Based on these findings, the intrinsic differences in baseline FAK signaling and the rapid regulation of FAK signaling following DPBS (Ca-Mg-) treatment may explain the differential responses between iMSCs and iPSCs to DPBS (Ca-Mg-) treatment **(Figure 5G)**.

### OCT4 knockdown in iPSCs: ITGA6 downregulation, FAK signaling activation, and establishing resistance to DPBS(Ca-Mg-)

The differences in FAK signaling expression levels and the response of FAK signaling to DPBS (Ca-Mg-) treatment can explain the variations in adhesion ability between iPSCs and iMSCs following DPBS (Ca-Mg-) treatment. To further investigate the reasons behind the differences in FAK signaling between iPSCs and iDCs, this study focused on two genes, OCT4 and ITGA6, identified through RNA-seq analysis and literature review. Pluripotency genes, such as OCT4, play a crucial role in the reprogramming and maintenance of pluripotency in stem cells, ensuring their ability to self-renew and differentiate into various cell types 50, 51. During the differentiation of PSCs into specific cell types, there is a concurrent downregulation of pluripotency genes (including OCT4) and the activation of differentiation-specific genes 50.

**Figure 7:**
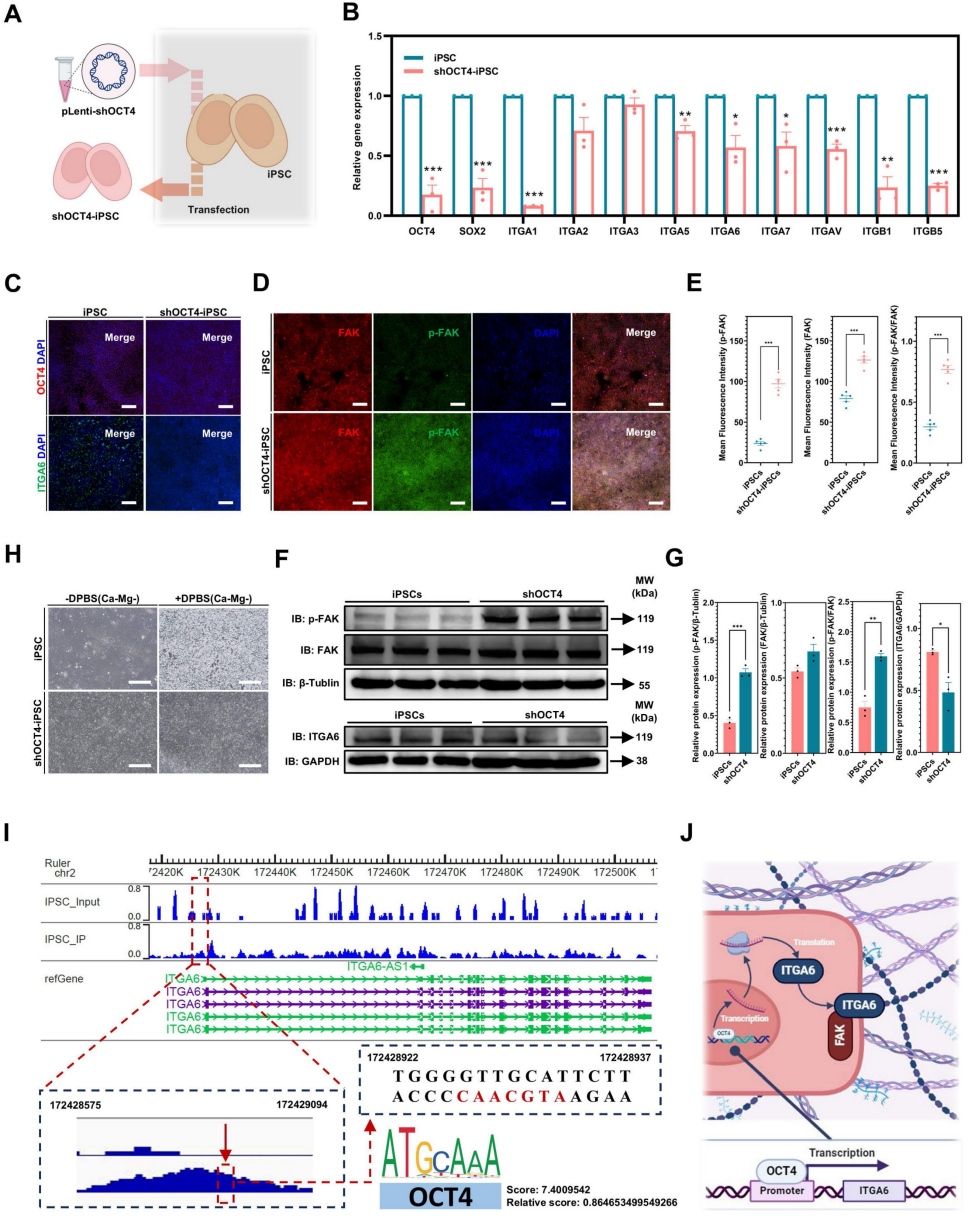
** Knockdown of OCT4 in iPSCs enhances their resistance to DPBS (Ca-Mg-) treatment.** A. Schematic diagram illustrating the knockdown of OCT4 in iPSCs using shOCT4; B. qPCR analysis showing the expression differences in common pluripotency genes and integrin genes between iPSCs and shOCT4-iPSCs. Data are derived from three independent experiments (n = 3) and are presented as mean ± SEM. Statistical analysis was performed using Student's t-test (*p < 0.05; **p < 0.01; ***p < 0.001); C. Immunofluorescence images of OCT4 and ITGA6 in iPSCs and shOCT4-iPSCs. Scale bars, 100 µm; D-E. Immunofluorescence images and quantification of p-FAK and FAK expression in iPSCs and shOCT4-iPSCs based on five independent experiments (n = 5). Data are presented as mean ± SEM, with group comparisons performed using Student's t-test (*p < 0.05; **p < 0.01; ***p < 0.001). Scale bars, 200 µm; F-G. Western blot analysis and quantification of p-FAK, FAK, and ITGA6 expression in iPSCs and shOCT4-iPSCs. Data are presented as mean ± SEM (n = 3), with component statistical comparisons performed using Student's t-test (*p < 0.05; **p < 0.01; ***p < 0.001); H. Bright-field images showing the morphology of iPSCs and shOCT4-iPSCs before and after DPBS (Ca-Mg-) treatment. Scale bars, 200 µm; I. The ChIP-seq peaks of OCT4 in iPSCs. Peaks represent regions with significant ChIP-seq enrichment, and the Y-axis shows ChIP-seq signal intensity (reads). X-axis represents genomic coordinates. The highlighted region corresponds to the binding site of OCT4 to the ITGA6 promoter region. For specific binding sites in this binding region, JASPAR was applied to predict the site; J. Schematic diagram showing the regulation of ITGA6 transcription and translation by OCT4.

Therefore, a lentiviral vector was used in this study to transduce shOCT4 into iPSCs, simulating the early downregulation of OCT4 during the differentiation of iPSCs into iDCs **(Figure 7A)**. The successful knockdown of OCT4 in iPSCs by shOCT4 was confirmed by qPCR and immunofluorescence results **(Figure 7B-C)**. Interestingly, the knockdown of OCT4 in iPSCs alone led to a significant downregulation of the pluripotency gene SOX2 and several common integrin genes, including ITGA6, ITGA1, ITGA3, ITGA5, ITGA7, ITGAV, ITGB1, and ITGB5 **(Figure 7B)**. Notably, among all the common integrin types analyzed, ITGA6 was the only one integrin showing a consistent trend of downregulation in both shOCT4-iPSCs and iMSCs compared to iPSCs. The downregulation of ITGA6 in shOCT4-iPSCs was further confirmed at the protein level through both immunofluorescence and Western blot analysis** (Figure 7C,F-G)**. Upon OCT4 knockdown, shOCT4-iPSCs exhibited a significant activation of FAK signaling, indicated by an increase in both p-FAK levels and the p-FAK/FAK ratio, which enhanced their resistance to DPBS (Ca-Mg-)** (Figure 7D-H)**. Unlike iPSCs, DPBS (Ca-Mg-) treatment for 30 min at 37°C did not result in the detachment of shOCT4-iPSCs **(Figure 7H)**.

OCT4 is a well-known transcription factor 51, and in this study, it was shown to be associated with ITGA6 expression. To explore whether OCT4 directly interacts with the promoter region of ITGA6, this study employed ChIP-seq to identify genome-wide binding sites of OCT4. ChIP-seq revealed 111,643 significant OCT4 binding peaks across the genome (p < 0.05), with 4,514 peaks located in promoter regions. As shown in **Figure 7I**, OCT4 was enriched in the promoter region of ITGA6 (binding peak region: chr2, 172428575-172429094). Further analysis using the JASPAR database predicted the precise binding motif within the identified ChIP-seq peak. JASPAR analysis found a recognizable OCT4 motif (5'-ATGCAAC-3', chr2, 172428922-172428937) within the OCT4-ITGA6 promoter binding region **(Figure 7I)**. This suggested that OCT4 may maintain ITGA6 expression by directly binding to the ITGA6 promoter and activating its transcription. When OCT4 expression is downregulated, ITGA6 expression is also reduced accordingly **(Figure 7J)**.

### ITGA6 knockdown and blocking in iPSCs: FAK signaling activation and establishing resistance to DPBS(Ca-Mg-)

**Figure 8:**
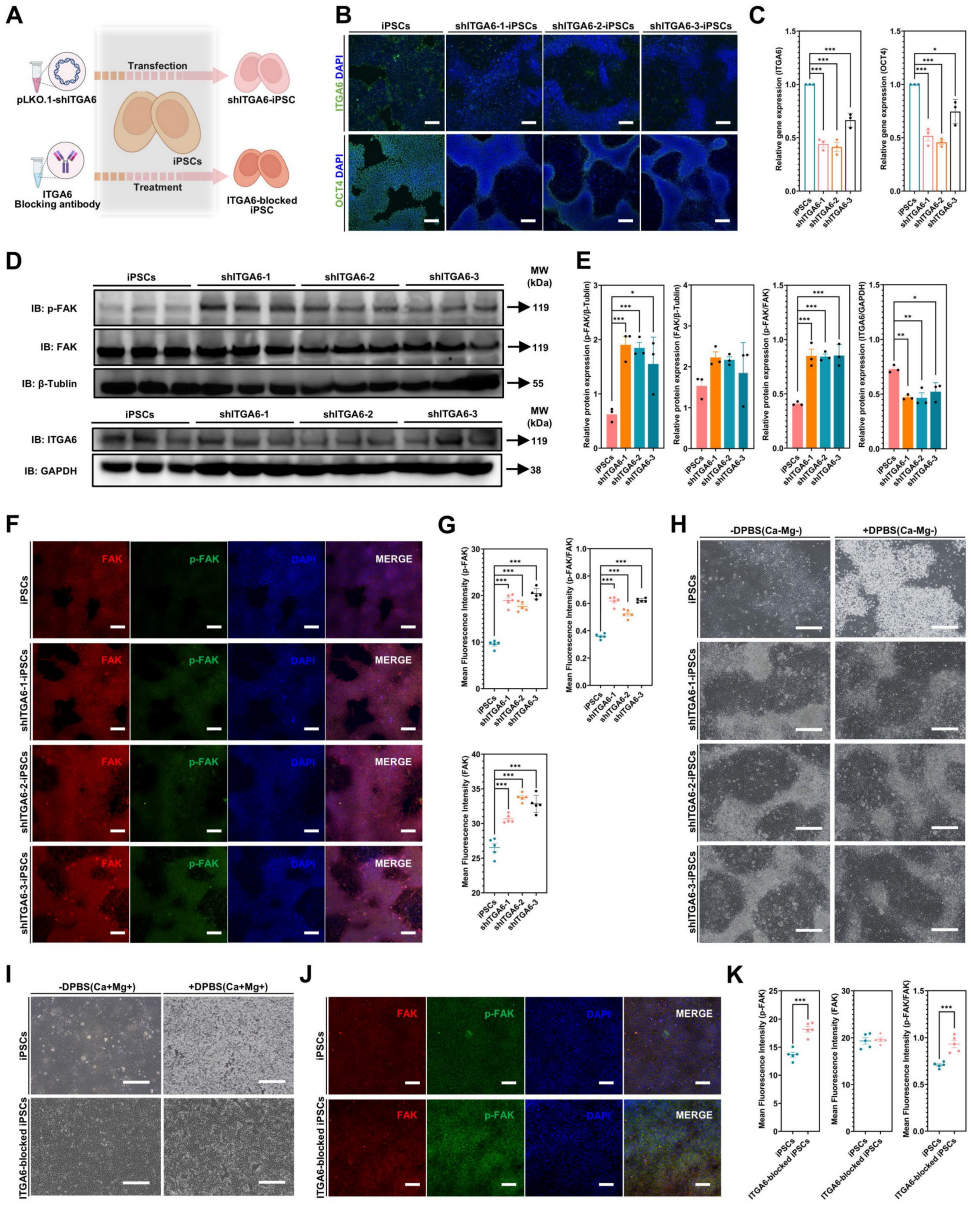
** Knockdown or blocking of ITGA6 in iPSCs enhances their resistance to DPBS (Ca-Mg-) treatment.** A. Schematic diagram illustrating the knockdown or blocking of ITGA6 in iPSCs; B. Immunofluorescence images of OCT4 and ITGA6 in iPSCs and shITGA6-iPSCs. Scale bars, 200 µm; C. qPCR analysis of OCT4 and ITGA6 gene expression in iPSCs and shITGA6-iPSCs. Data from three independent experiments (n = 3) are presented as mean ± SEM, with statistical analysis performed using one-way ANOVA (*p < 0.05; **p < 0.01; ***p < 0.001); D-E. Western blot analysis and quantification of p-FAK, FAK, and ITGA6 expression in iPSCs and shITGA6-iPSCs. Data (n = 3) are presented as mean ± SEM, with component statistical comparisons performed using one-way ANOVA; F-G. Immunofluorescence images and quantification of p-FAK and FAK expression in iPSCs and shITGA6-iPSCs, based on five independent experiments (n = 5). Data are presented as mean ± SEM, and statistical comparison was performed using one-way ANOVA (***p < 0.001); H. Bright-field images showing iPSCs and shITGA6-iPSCs before and after DPBS (Ca-Mg-) treatment. Scale bars, 200 µm; I. Bright-field images showing iPSCs and ITGA6-blocked iPSCs before and after DPBS (Ca-Mg-) treatment. Scale bars, 200 µm; J-K. Immunofluorescence images and quantification of p-FAK and FAK expression in iPSCs and shITGA6-iPSCs, based on five independent experiments (n = 5). Data are presented as mean ± SEM, and statistical comparison was performed using Student's t-test (***p < 0.001).

Furthermore, lentiviral vectors were used to transduce shITGA6 into iPSCs to identify whether ITGA6 signaling is the key mediator of OCT4 regulation of the FAK pathway** (Figure 8A)**. Immunofluorescence and WB results showed that in all three ITGA6 knockdown iPSC lines (shITGA6-1-iPSCs, shITGA6-2-iPSCs, and shITGA6-3-iPSCs), ITGA6 expression was significantly downregulated, while p-FAK and p-FAK/FAK was significantly upregulated **(Figure 8B-G)**. After ITGA6 knockdown, DPBS (Ca-Mg-) treatment (37 °C, 30 min) did not result in cell detachment **(Figure 8H)**.

Notably, OCT4 downregulation was observed in all three ITGA6 knockdown groups **(Figure 8B,E)**. The downregulation of the pluripotency gene OCT4 is a typical marker of PSC differentiation. Our results suggested that ITGA6 appeared to play an important role in maintaining PSC pluripotency. Furthermore, this study utilized an ITGA6 blocking antibody (GoH3, 40 µg/ml) to directly inhibit ITGA6 activity and examine potential changes in FAK signaling and the response to DPBS (Ca-Mg-) treatment **(Figure 8A)**. As shown in **Figure 8I**, after 24 hours of treatment with the ITGA6 blocking antibody, most cells exhibited an increased resistance to DPBS (Ca-Mg-) treatment, remaining adherent post-treatment. On the other hand, immunostaining results indicated that ITGA6 blocking antibody treatment significantly upregulated p-FAK and the p-FAK/FAK ratio in iPSCs **(Figure 8J-K)**. This aligns with the findings observed from directly knocking down ITGA6 in iPSCs.

Additionally, this study examined the impact of FAK signaling on the expression of the pluripotency gene OCT4. The iPSCs were treated with different concentrations of FAK inhibitor (FAKi) to suppress FAK signaling in these cells **(Figure S7A)**. As shown in **Figure S7B**, FAKi treatment (100 nM and 1 µM) significantly upregulated OCT4 gene expression in iPSC. Subsequently, iPSCs were treated again with 100 nM FAKi, and OCT4 level were measured via immunofluorescence. The results indicated that FAKi effectively upregulated the protein level of OCT4 **(Figure S7C-D)**. These results suggested a complex regulatory network between OCT4, ITGA6, and FAK signaling in PSCs. OCT4 and ITGA6 appear to be involved in a positive feedback loop, with ITGA6 inhibiting FAK signaling **(Figure S7E)**. This weakened FAK signaling contributes to the detachment response to BBS (Ca-Mg-) of PSCs. Additionally, a negative feedback mechanism exists for FAK signaling, where inhibition of FAK signaling leads to increased expression of the pluripotency gene OCT4 in iPSCs **(Figure S7E)**. This regulation suggests a potential link between the cell adhesion signaling pathway (mediated by FAK and integrins) and the maintenance of stem cell pluripotency (indicated by OCT4 levels). The upregulation of OCT4 may indicate an adaptive response of iPSCs to the altered signaling environment, potentially enhancing their stemness when FAK activity is inhibited.

## Discussion

In this study, we developed a novel approach for removing residual PSCs using BSS(Ca-Mg-), capitalizing on the distinct responses of PSCs and iDCs to BSS(Ca-Mg-) treatment. Unlike previous methods that triggered PSC death through various mechanisms 13, 15-17, BSS(Ca-Mg-) selectively induced rapid detachment of PSCs without affecting the adhesion of the iDCs tested. Although further validation with additional iDC types is needed, the stability of iMSCs characteristics after BSS(Ca-Mg-) treatment supports the safety of this method. Moreover, our findings reveal a complex signaling network in PSCs involving ITGA6, OCT4, and FAK signaling, which contributes to the differential response between PSCs and iDCs. This innovative strategy of pre-treating iDCs *in vitro* could help reduce the risk of teratoma formation in iDC-based therapies.

We first address the efficacy and safety of BSS(Ca-Mg-) treatment. In our co-culture model of PSCs and iDCs, BSS(Ca-Mg-) successfully cleared residual PSCs in at least four types of iDCs, including iMSCs, iOBs, iFCs, and iEPCs. However, while no teratomas were observed *in vivo* following BSS(Ca-Mg-) treatment, our *in vitro* data did not demonstrate complete removal of PSCs. The occurrence of teratomas remains unpredictable and is highly dependent on the number of residual PSCs introduced 52, 53. Studies in immunodeficient mice have shown that at least 1 x 10^5^ hESCs injected into the myocardium and 1 x 10^4^ hESCs injected into skeletal muscle are required to form teratomas 52. Therefore, while complete clearance may not be achieved, we anticipate that BSS(Ca-Mg-) treatment could reduce the risk of teratoma formation by lowering the number of residual PSCs in iDC injections. Additionally, PBS and DPBS are commonly used reagents in cell culture, being non-toxic to most cells and suitable for various applications such as cell washing, cell or tissue sample transport, cell dilution for counting, and reagent preparation 54, 55. In our experiments, we did not observe any toxic effects of DPBS(Ca-Mg-) on iMSCs. The safety profile of BSS(Ca-Mg-) supports its potential use either as a standalone method or in combination with other strategies for residual PSC clearance. Future studies should explore a wider array of combinations involving BSS(Ca-Mg-) and different iDCs to thoroughly evaluate its applicability, efficacy, and safety.

In the mechanisms section, we have expanded upon the important findings by Villa-Diaz *et al.* 56 and conducted further investigations. Our results reveal a positive feedback regulation between the pluripotency gene OCT4 and ITGA6, suggesting that OCT4 regulation of ITGA6 may be related to its direct interaction with the ITGA6 promoter. The transcription factor OCT4 is highly expressed in PSCs and plays a critical role in the induction and maintenance of pluripotency 57. The direct positive regulation of ITGA6 transcription by Oct4 accounts for ITGA6 being one of the most frequently expressed integrins in PSCs 56. However, the mechanisms by which ITGA6 contributes to the upregulation of Oct4 expression remain unclear and require further investigation.

Additionally, both OCT4 and ITGA6 exhibit negative regulation of FAK signaling, which aligns with previous studies reporting low levels of FAK phosphorylation in undifferentiated PSCs 56. FAK, a non-receptor tyrosine kinase 58, relies on phosphorylation at the Tyr-397 site for the assembly of focal adhesions (FAs), a process that is driven by interactions between the extracellular matrix (ECM) and integrins 59, 60. And FA-based cell-ECM interactions are essential for cellular anchoring 61, 62. Therefore, the lower p-FAK levels in PSCs themselves and after BSS (Ca-Mg-) treatment compared to iDCs may be the reason why BSS (Ca-Mg-) causes loss of adhesion in PSCs (but not iDCs). This was confirmed by subsequent experiments. On the one hand, either knockdown of OCT4 (one of the pluripotency markers in PSCs) or ITGA6 in PSCs increased cellular resistance to BSS (Ca-Mg-) while up-regulating FAK phosphorylation and manifested as a maintenance of the adhesion state. On the other hand, inhibition of FAK phosphorylation in iMSCs (with lower expression of OCT4 and ITGA6 compared to PSCs) induced loss of adhesion after BSS (Ca-Mg-) treatment.

Interestingly, inhibition of FAK signaling in PSCs led to a further upregulation of the expression level of OCT4, which is expressed at high levels in PSCs, suggesting the existence of a complex regulatory network of OCT4, ITGA6, and FAK signaling in the domains of pluripotency maintenance, differentiation, and cell adhesion in PSCs. The role of ITGA6 in maintaining OCT4 expression may be partly attributed to ITGA6-mediated downregulation of FAK phosphorylation. Therefore, further investigation of the process and expression of these signals may help to reveal the mechanisms involved in multiple biological processes of PSCs and further develop strategies that can completely remove residual PSCs from iDCs.

This study has certain limitations. First, although we employed a co-culture system of PSCs and iDCs to mimic the presence of residual pluripotent stem cells during differentiation, this model may not fully replicate the *in vivo* differentiation environment. Second, we only evaluated the ability of BSS(Ca-Mg-) to remove residual PSCs in four types of iDCs (iMSCs, iFCs, iOBs, and iEPCs). Whether this approach is effective for other PSC-derived cell types remains unexplored. Finally, we have not directly compared the clearance efficiency of this method with other reported strategies. Future studies will focus on addressing these limitations, further optimizing the approach, and clarifying its potential clinical applications.

## Conclusion

In conclusion, we have proposed a novel strategy for removing residual PSCs in iDCs using BSS(Ca-Mg-), and validated its effectiveness across various iDCs, including iMSCs, iOBs, iFCs, and iEPCs. Treatment with BSS(Ca-Mg-) rapidly and efficiently induced detachment of iPSCs without causing damage to iDCs, and significantly inhibited teratoma formation in immunodeficient animal models. This effect is closely linked to the differential regulation of OCT4, ITGA6, and FAK signaling between PSCs and iDCs. This innovative approach provides a safe strategy for future research and clinical translation of iDCs. Further testing of this method across a broader range of BSS(Ca-Mg-) conditions and iDCs could facilitate its wider application and help overcome key challenges in the clinical translation of PSC-based therapies.

## Supplementary Material

Supplementary figures and tables.
