# Supplementary material for: FAK signaling suppression by OCT4-ITGA6 mediates the effectively removal of residual pluripotent stem cells and enhances application safety
Source: Theranostics. 2025 Jun 12;15(14):7127–53. doi: 10.7150/thno.111198 (PMC12204078; doi:10.7150/thno.111198)
Supplement: Supplementary file 1 — Supplementary figures and tables. [file thnov15p7127s1.pdf]

Supplementary Materials for

- **FAK signaling suppression by OCT4-ITGA6 mediates the effectively removal of residual pluripotent stem cells and enhances application safety**

Wenpeng Song, *et al.*

*Yuchun Gu. Email: ycgu@pku.edu.cn

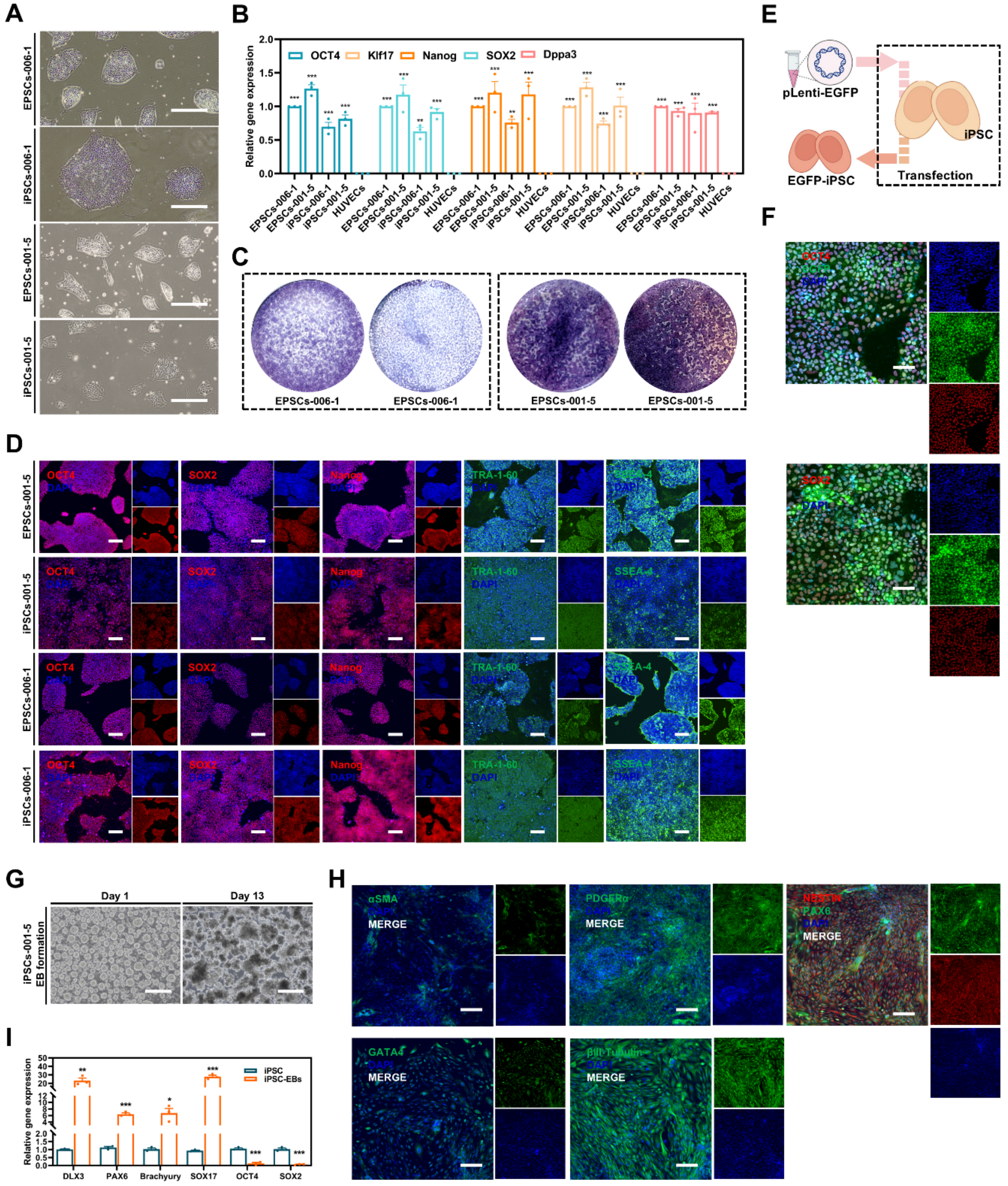

Figure S1. Identification of PSCs. A. Bright-field images of iPSCs-001-5, EPSCs-001-5, iPSCs-006-1, and EPSCs-006-1. Scale bars, 100 μm; B. qPCR analysis showing the differential expression of pluripotency genes (OCT4, SOX2, Nanog, Klf17, Dppa3) between PSCs and the negative control HUVECs. Data are from three independent experiments (n = 3), presented as mean ± SEM, and statistically analyzed using one-way ANOVA. Comparison with HUVECs: *$p < 0.05$; **$p < 0.01$; ***$p < 0.001$; C. Crystal violet staining images of iPSCs-001-5, EPSCs-001-5, iPSCs-006-1, and EPSCs-006-1; D. Fluorescence images showing the protein expression

of pluripotency markers in the four PSC lines. Scale bars, 200 μm; E. Schematic representation of the construction of EGFP-iPSCs; F. Fluorescence images displaying the expression of EGFP, OCT4, and SOX2 in EGFP-iPSCs. Scale bars, 100 μm; G. Bright-field images of EBs formed by iPSCs. Scale bars, 200 μm; H. Fluorescence images showing the expression of three germ layer protein markers in EBs after attachment. Scale bars, 200 μm; I. qPCR analysis of pluripotency genes and three germ layer differentiation marker genes in iPSCs and iPSC-derived EBs. Data are from three independent experiments (n = 3), presented as mean ± SEM, and statistically analyzed using Student's t-test. *$p < 0.05$; **$p < 0.01$; ***$p < 0.001$.

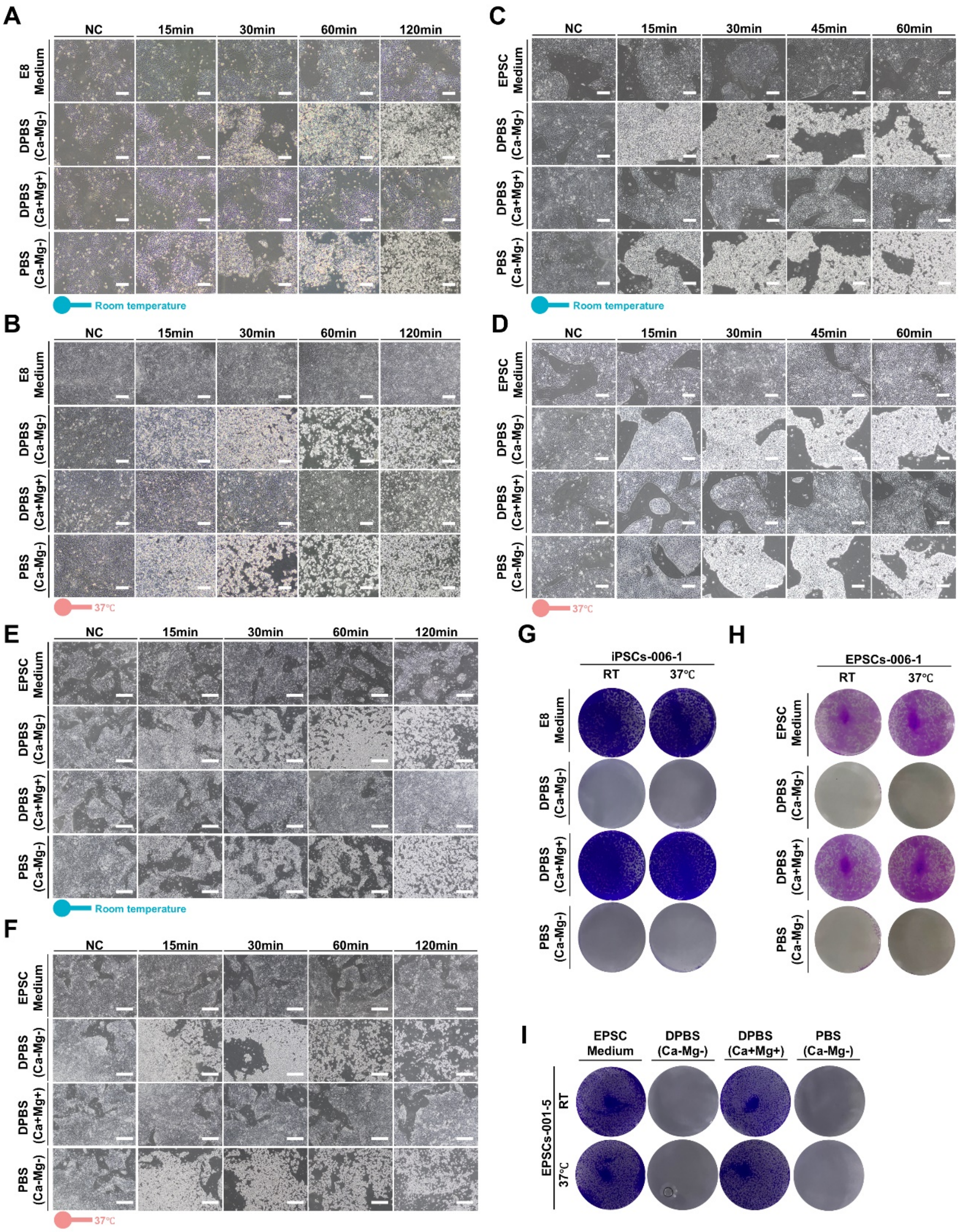

Figure S2. BSS (Ca-Mg-) treatment causes detachment of multiple PSCs. A-B. Bright-field images of iPSCs-006-1 before and after treatment with BSS (Ca-Mg-) at 37° C and room temperature. Scale bars, 50 µm; C-D. Bright-field images of EPSCs-006-1 before and after treatment with BSS (Ca-Mg-) at 37° C and room temperature. Scale bars, 50 µm; E-F. Bright-field images of EPSCs-001-5 before and after treatment with BSS (Ca-Mg-) at 37° C and room

temperature. Scale bars, 200 μm; G. Crystal violet staining image of iPSCs-006-1 after BSS (Ca-Mg-) treatment; H. Crystal violet staining image of EPSCs-006-1 after BSS (Ca-Mg-) treatment; I. Crystal violet staining image of EPSCs-001-5 after BSS (Ca-Mg-) treatment.

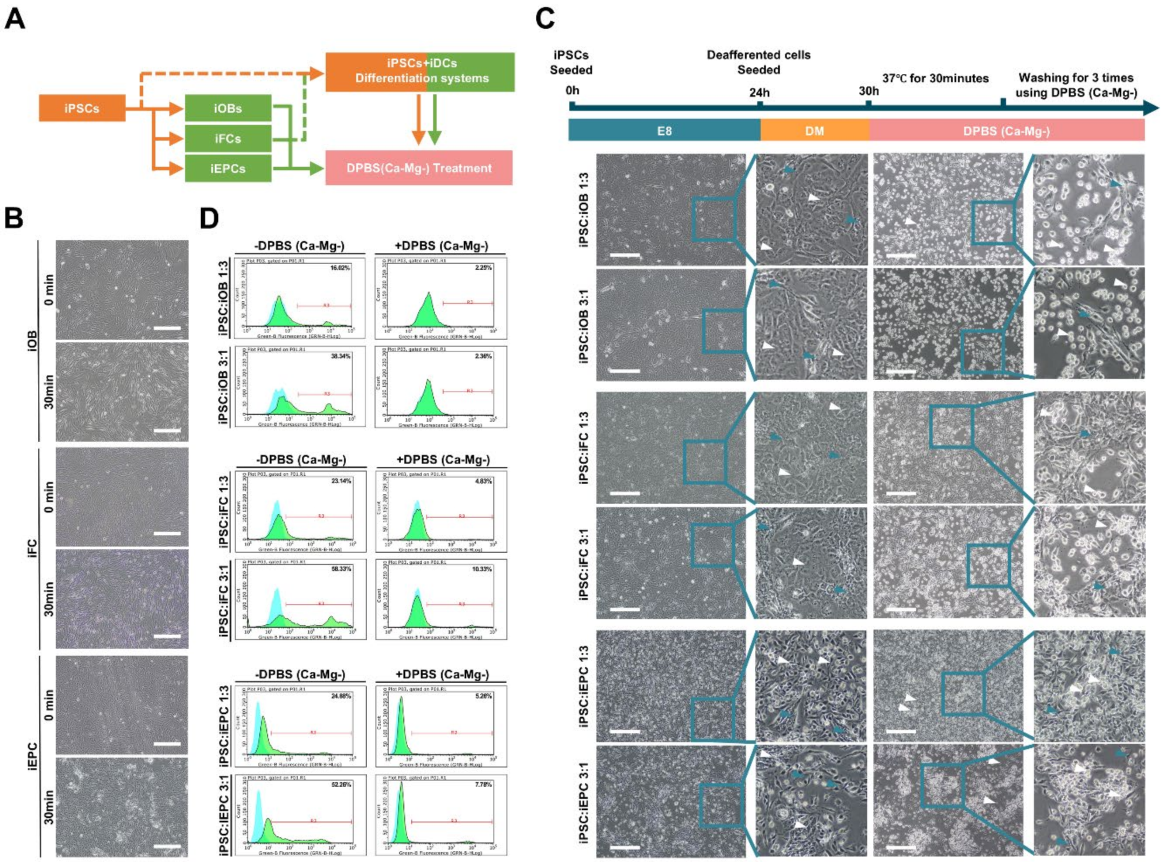

Figure S3. Establishment of iPSCs and iDC co-culture systems and DPBS (Ca-Mg-) treatment. A. Schematic diagram of the co-culture system of iPSCs with iFCs, iOBs, and iEPCs, and the subsequent DPBS (Ca-Mg-) treatment; B. Bright-field images of iOBs, iFCs, and iEPCs before and after DPBS (Ca-Mg-) treatment (37° C, 30 min). White arrows indicate iPSCs, and blue arrows indicate iDCs. Scale bars, 200 μm; C. Bright-field images of the iFCs, iOBs, and iEPCs co-culture system and after DPBS (Ca-Mg-) treatment. Scale bars, 200 μm; D. Flow cytometry analysis showing the percentage of EGFP-positive cells among adherent cells before and after DPBS (Ca-Mg-) treatment.

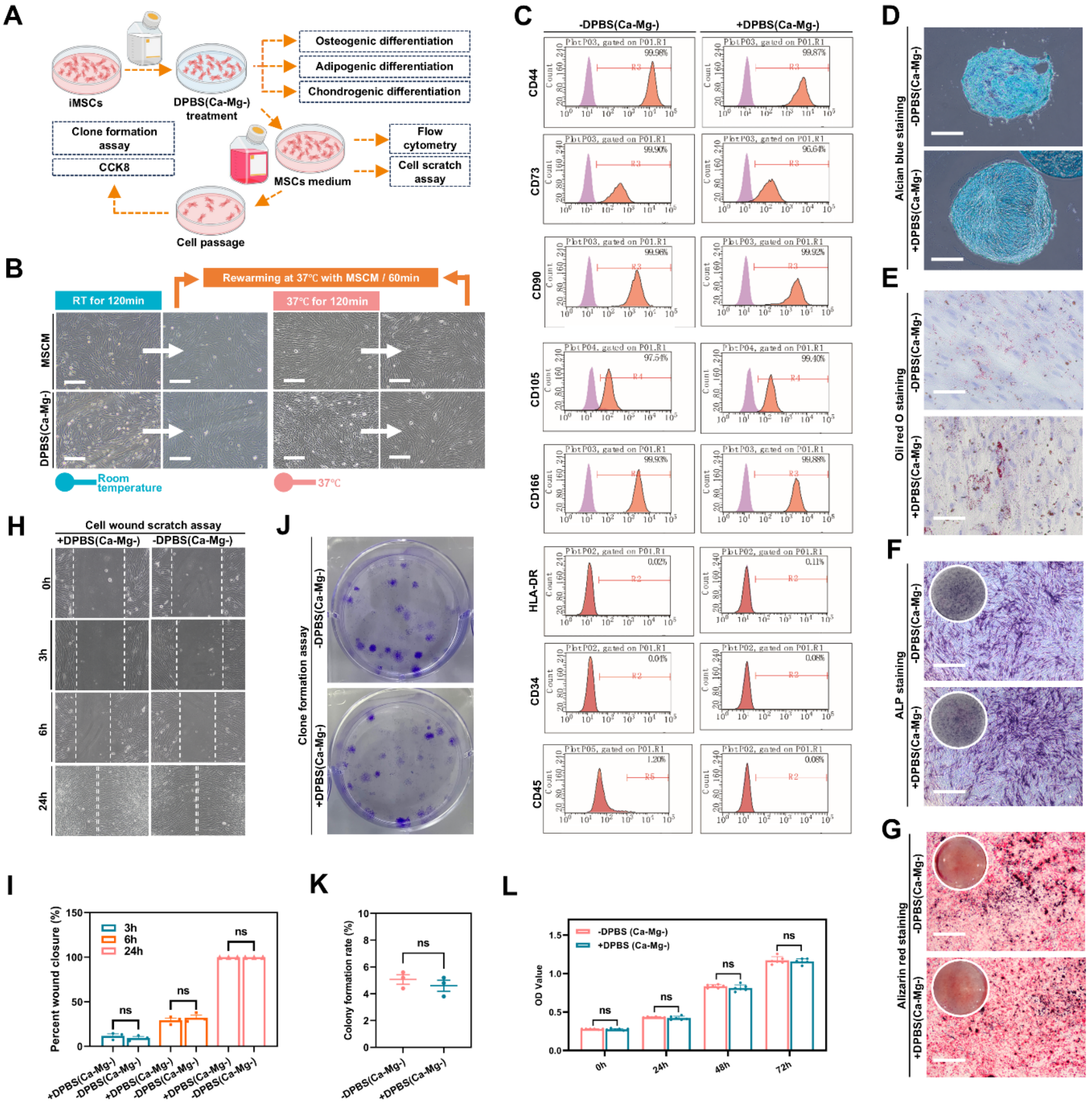

Figure S4. DPBS (Ca-Mg-) short-term treatment does not affect iMSCs characteristics. A. Schematic diagram of iMSCs characteristic detection before and after DPBS (Ca-Mg-) treatment; B. Bright-field images of iMSCs cultured with MSCM (37° C) after DPBS (Ca-Mg-) treatment at 37° C and room temperature. Scale bars, 200 µm; C. Flow cytometry analysis of surface markers (CD44, CD73, CD90, CD105, CD166, HLA-DR, CD34, and CD45) expression in iMSCs before and after DPBS (Ca-Mg-) treatment; D. Alcian blue staining to assess the chondrogenic differentiation ability of iMSCs before and after DPBS (Ca-Mg-) treatment. Scale bars, 200 µm; E. Oil Red O staining to assess the adipogenic differentiation ability of iMSCs before and after DPBS (Ca-Mg-) treatment. Scale bars, 50 µm; F-G. ALP staining and alizarin red staining to assess the osteogenic differentiation ability of iMSCs before and after DPBS (Ca-Mg-) treatment. Scale bars, 200 µm; H-I. Wound healing assay and subsequent quantification to

evaluate the cell migration ability of iMSCs before and after DPBS (Ca-Mg-) treatment. Data from three independent experiments (n = 3) are presented as mean ± SEM, and statistical analysis was performed using two-way ANOVA. ns $p > 0.05$, *$p < 0.05$; **$p < 0.01$; ***$p < 0.001$. J-K. Colony formation assay and subsequent quantification to evaluate the self-renewal ability of iMSCs before and after DPBS (Ca-Mg-) treatment. Data from three independent experiments (n = 3) are presented as mean ± SEM, and statistical analysis was performed using Student's t-test. ns $p > 0.05$, *$p < 0.05$; **$p < 0.01$; ***$p < 0.001$; L. CCK8 assay to assess the cell proliferation ability of iMSCs before and after DPBS (Ca-Mg-) treatment. Data (n = 6) are presented as mean ± SEM, and statistical analysis was performed using two-way ANOVA. ns $p > 0.05$; *$p < 0.05$; **$p < 0.01$; ***$p < 0.001$.

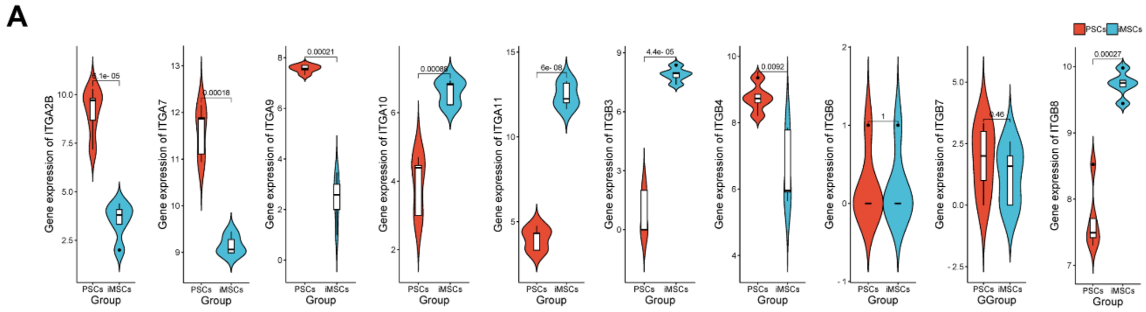

Figure S5. A. Violin plot comparing the expression levels of common integrins in PSCs and iMSCs. The p-values from the Wilcoxon test are shown in the figure.

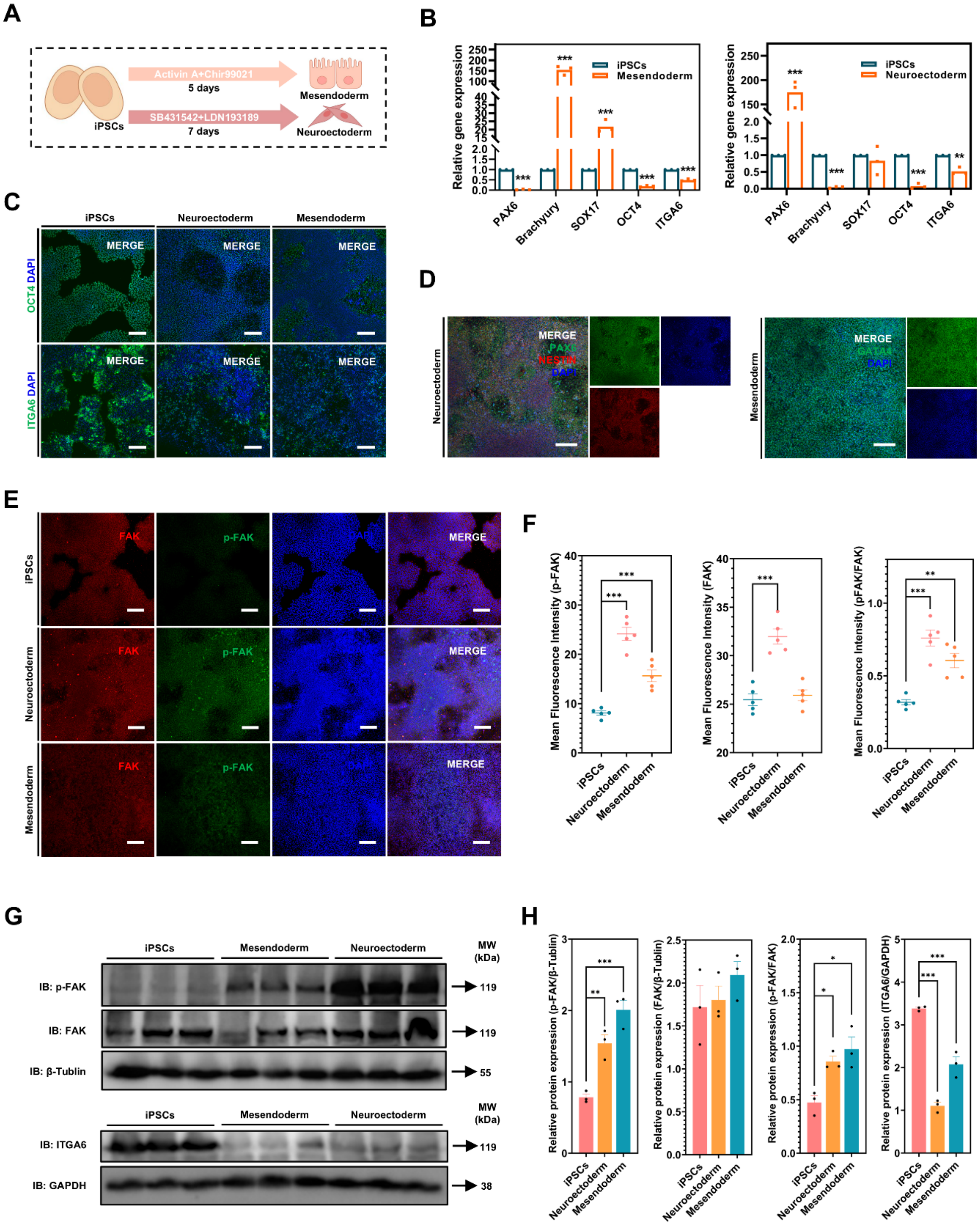

Figure S6. Altered OCT4, ITGA6, and FAK signaling in iPSCs after differentiation into neuroectoderm and mesendoderm. A. Schematic diagram showing the differentiation of iPSCs into neuroectoderm and mesendoderm; B. qPCR analysis showing changes in differentiation markers and pluripotency markers after iPSCs differentiation into neuroectoderm and mesendoderm; C. Fluorescence images showing the protein expression of OCT4 and ITGA6

after iPSCs differentiation into neuroectoderm and mesendoderm. Scale bars, 200 μm; D. Fluorescence images showing the expression of differentiation markers after iPSCs differentiation into neuroectoderm and mesendoderm. Scale bars, 100 μm; E-F. Fluorescence images showing the expression and quantification of p-FAK and FAK after iPSCs differentiation into neuroectoderm and mesendoderm. Scale bars, 200 μm. Data ($n = 5$) are presented as mean ± SEM and analyzed using one-way ANOVA. *$p < 0.05$; **$p < 0.01$; ***$p < 0.001$; G-H. Western blot results and quantification of p-FAK, FAK, and ITGA6 after iPSCs differentiation into neuroectoderm and mesendoderm. Data ($n = 3$) are presented as mean ± SEM and analyzed using one-way ANOVA. *$p < 0.05$; **$p < 0.01$; ***$p < 0.001$.

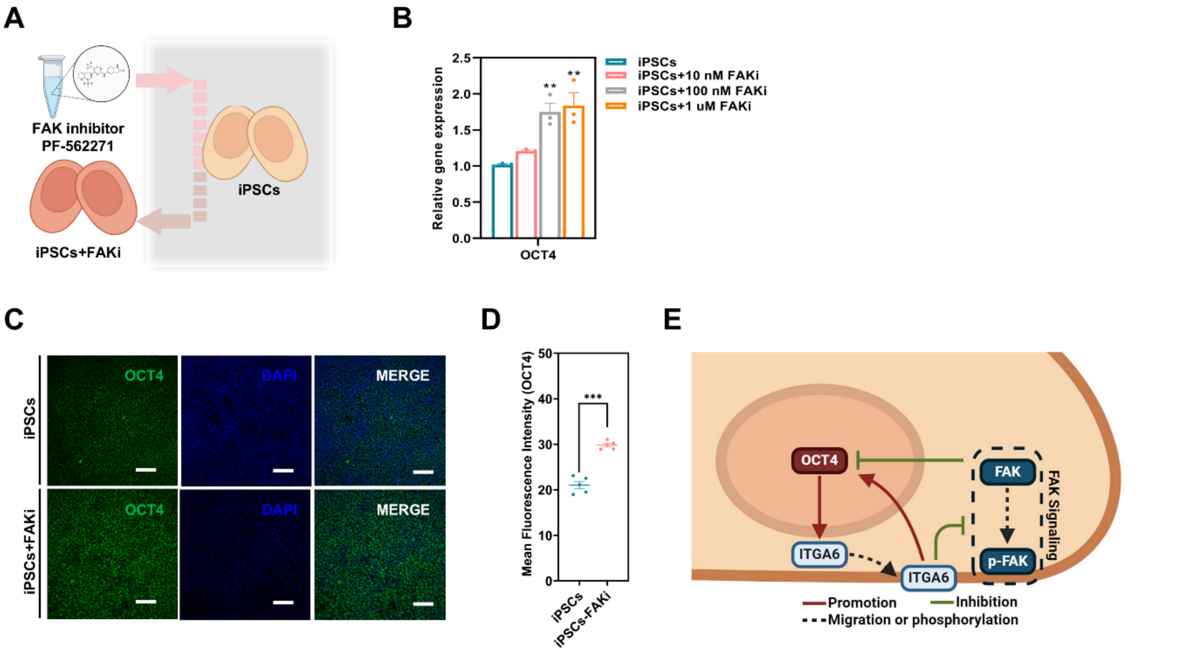

Figure S7. Inhibition of FAK signaling upregulated OCT4 expression in iPSCs. A. Schematic diagram illustrating the strategy for inhibiting FAK signaling in iMSCs using a PF-562271; B. qPCR analysis of the expression levels of OCT4 in FAKi-treated iPSCs. Data are presented as mean ± SEM from three independent experiments (n = 3), with statistical significance determined using one-way ANOVA (*p < 0.05; **p < 0.01; ***p < 0.001); C-D. Immunofluorescence images and quantitative analysis of p-FAK and FAK in iPSCs treated with FAKi. Quantification is based on five independent experiments (n = 5), with data presented as mean ± SEM and analyzed using one-way ANOVA, *p < 0.05; **p < 0.01; ***p < 0.001. Scale bars, 100 μm; E. Schematic diagram of regulation between OCT4, ITGA6 and FAK signaling in iPSCs.

Gene Symbol	Sequence (5'->3')	
OCT4	Forward primer	GTGTTCAGCCAAAAGACCATCT
	Reverse primer	GGCCTGCATGAGGGTTTCT
SOX2	Forward primer	GGGAAATGGGAGGGGTGCAAAAGAGG
	Reverse primer	TTGCGTGAGTGTGGATGGGATTGGTG
Nanog	Forward primer	TTTGTGGGCCTGAAGAAAACT
	Reverse primer	AGGGCTGTCCTGAATAAGCAG
KLF17	Forward primer	GCTGCCCAGGATAACGAGAAC
	Reverse primer	ATCTCTGCGCTGTGAGGAAAG
Dppa3	Forward primer	TTAATCCAACCTACATCCCAGGG
	Reverse primer	AGGGGAAACAGATTCGCTACTA
ITGA1	Forward primer	GGTTACCCTGTGCTGTACCCAA
	Reverse primer	TGCCTCGTTTGAGATGGTCA
ITGA2	Forward primer	TCTGAGACTGCCAAGGTCTTCA
	Reverse primer	CAGCTGGTATTTGTCGGACATC
ITGA3	Forward primer	GAACCCCTTCAAACGGAACC
	Reverse primer	ACCTCAAAGGCGATGAGCAG
ITGA5	Forward primer	AGATCCTGAAATGCCCGGA
	Reverse primer	CAGACTCGGAAATGCAACTGC
ITGA6	Forward primer	GTTTGATAACGATGCTGACCCC
	Reverse primer	TGAGCACATGTCACGACCTTG
ITGA7	Forward primer	AACCTGGAAGAACCCAAGCAC
	Reverse primer	TGACATTTTCCTGGAGCTGGA
ITGAV	Forward primer	TGACATTTTCCTGGAGCTGGA
	Reverse primer	TCTCTGACTGCTGGTGCACACT
ITGB1	Forward primer	ATGCCATCATGCAAGTTGCA
	Reverse primer	CCCATCTCCAGCAAAGTGAAAC
ITGB5	Forward primer	ACCAAGAGAGATTGCGTCGAGT
	Reverse primer	CAGCCTCCTGGTCATCTTTCA
GAPDH	Forward primer	GGAGCGAGATCCCTCCAAAAT
	Reverse primer	GGCTGTTGTCATACTTCTCATGG

Table S1. The primer sequences applied in this study.

Antibody	Item number	Company	Location
Anti-OCT4 antibody	ab19857	Abcam	Cambridge, UK
Anti-SOX2 antibody	ab97959	Abcam	Cambridge, UK
Anti-Nanog antibody	ab218524	Abcam	Cambridge, UK
Anti-TRA-1-60 antibody	ab16288	Abcam	Cambridge, UK
Anti-SSEA-4 antibody	MC-813-70	Invitrogen	California, USA
Anti- α SMA antibody	ab7817	Abcam	Cambridge, UK
Anti-GATA4 antibody	WL01293	VANCL Biotech	Jiangsu, China
Anti-PDGFR α antibody	Ab203491	Abcam	Cambridge, UK
Anti- β III-Tubulin antibody	ab18207	Abcam	Cambridge, UK
Anti-PAX6 antibody	ab5790	Abcam	Cambridge, UK
Anti-NESTIN antibody	ab22035	Abcam	Cambridge, UK
Anti-FAK (phospho Y397) antibody	ab81298	Abcam	Cambridge, UK
Anti-FAK antibody	66258-1-Ig	Proteintech	Illinois, USA
Anti-ITGA6 antibody	27189-1-AP	Proteintech	Illinois, USA
Anti-CD44 antibody	338808	Biolegend	California, USA
Anti-CD73 antibody	344016	Biolegend	California, USA
Anti-CD90 antibody	328108	Biolegend	California, USA
Anti-CD105 antibody	560819	BD Biosciences	New Jersey, USA
Anti-CD166 antibody	343904	Biolegend	California, USA
Anti-CD34 antibody	555821	BD Biosciences	New Jersey, USA
Anti-CD45 antibody	555482	BD Biosciences	New Jersey, USA
Anti-HLA-DR antibody	555811	BD Biosciences	New Jersey, USA

Table S2. The primary antibody applied in this study.

Figure	Statistical methods	Groups	p-value
**Fig. 2E**	Two-way ANOVA	iPSC:iMSC 1:3 -DPBS(Ca-Mg-) vs. iPSC:iMSC 1:3 +DPBS(Ca-Mg-)	$p < 0.0001$
		iPSC:iMSC 3:1 -DPBS(Ca-Mg-) vs. iPSC:iMSC 3:1 +DPBS(Ca-Mg-)	$p < 0.0001$
**Fig. 2F**	Two-way ANOVA	iPSCs vs. iMSCs	$p=0.0001$
		iPSCs vs. iPSC:iMSC 1:3 -DPBS(Ca-Mg-)	$p=0.1439$
		iPSCs vs. iPSC:iMSC 1:3 +DPBS(Ca-Mg-)	$p=0.0001$
		iPSCs vs. iPSC:iMSC 3:1 -DPBS(Ca-Mg-)	$p=0.0009$
		iPSCs vs. iPSC:iMSC 3:1 +DPBS(Ca-Mg-)	$p=0.0001$
		iMSCs vs. iPSC:iMSC 1:3 -DPBS(Ca-Mg-)	$p=0.0062$
		iMSCs vs. iPSC:iMSC 1:3 +DPBS(Ca-Mg-)	$p>0.9999$
		iMSCs vs. iPSC:iMSC 3:1 -DPBS(Ca-Mg-)	$p=0.6932$
		iMSCs vs. iPSC:iMSC 3:1 +DPBS(Ca-Mg-)	$p>0.9999$
**Fig. 2G**	One-way ANOVA	OCT4: iPSCs vs. iMSCs	$p<0.0001$
		OCT4: iPSCs vs. iPSC:iMSC 1:3 -DPBS(Ca-Mg-)	$p<0.0001$
		OCT4: iPSCs vs. iPSC:iMSC 3:1 -DPBS(Ca-Mg-)	$p=0.0052$
		OCT4: iPSCs vs. iPSC:iMSC 1:3 +DPBS(Ca-Mg-)	$p<0.0001$
		OCT4: iPSCs vs. iPSC:iMSC 3:1 +DPBS(Ca-Mg-)	$p<0.0001$
		OCT4: iMSCs vs. iPSC:iMSC 1:3 +DPBS(Ca-Mg-)	$p=0.8844$
		OCT4: iMSCs vs. iPSC:iMSC 3:1 +DPBS(Ca-Mg-)	$p=0.0641$
		SOX2: iPSCs vs. iMSCs	$p<0.0001$
		SOX2: iPSCs vs. iPSC:iMSC 1:3 -DPBS(Ca-Mg-)	$p<0.0001$
		SOX2: iPSCs vs. iPSC:iMSC 3:1 -DPBS(Ca-Mg-)	$p=0.0002$
		SOX2: iPSCs vs. iPSC:iMSC 1:3 +DPBS(Ca-Mg-)	$p<0.0001$
		SOX2: iPSCs vs. iPSC:iMSC 3:1 +DPBS(Ca-Mg-)	$p<0.0001$
		SOX2: iMSCs vs. iPSC:iMSC 1:3 +DPBS(Ca-Mg-)	$p>0.9999$
		SOX2: iMSCs vs. iPSC:iMSC 3:1 +DPBS(Ca-Mg-)	$p=0.9973$
		Nanog: iPSCs vs. iMSCs	$p<0.0001$
		Nanog: iPSCs vs. iPSC:iMSC 1:3 -DPBS(Ca-Mg-)	$p<0.0001$
		Nanog: iPSCs vs. iPSC:iMSC 3:1 -DPBS(Ca-Mg-)	$p<0.0001$
		Nanog: iPSCs vs. iPSC:iMSC 1:3 +DPBS(Ca-Mg-)	$p<0.0001$
		Nanog: iPSCs vs. iPSC:iMSC 3:1 +DPBS(Ca-Mg-)	$p<0.0001$
		Nanog: iMSCs vs. iPSC:iMSC 1:3 +DPBS(Ca-Mg-)	$p>0.9999$
		Nanog: iMSCs vs. iPSC:iMSC 3:1 +DPBS(Ca-Mg-)	$p=0.9944$
**Fig. 5A**	Student's t-test	OCT4: iPSCs vs. iMSCs	$p=0.000028$
		SOX2: iPSCs vs. iMSCs	$p=0.000014$

		ITGA1: iPSCs vs. iMSCs	p=0.000835
		ITGA2: iPSCs vs. iMSCs	p=0.001296
		ITGA3: iPSCs vs. iMSCs	p=0.00277
		ITGA5: iPSCs vs. iMSCs	p=0.00057
		ITGA6: iPSCs vs. iMSCs	p=0.005573
		ITGA7: iPSCs vs. iMSCs	p=0.003685
		ITGAV: iPSCs vs. iMSCs	p=0.001086
		ITGB1: iPSCs vs. iMSCs	p=0.009233
		ITGB5: iPSCs vs. iMSCs	p=0.245077
**Fig. 5D**	One-way ANOVA	pFAK: iMSCs vs. iMSCs+DPBS(Ca-Mg-)	p<0.0001
		pFAK: iMSCs vs. iPSCs	p=0.0001
		pFAK: iMSCs vs. iPSCs+DPBS(Ca-Mg-)	p<0.0001
		pFAK: iMSCs+DPBS(Ca-Mg-) vs. iPSCs	p=0.9961
		pFAK:iMSCs+DPBS(Ca-Mg-) vs. iPSCs+DPBS(Ca-Mg-)	p=0.0003
		pFAK: iPSCs vs. iPSCs+DPBS(Ca-Mg-)	p=0.0002
		FAK: iMSCs vs. iMSCs+DPBS(Ca-Mg-)	p=0.9867
		FAK: iMSCs vs. iPSCs	p<0.0001
		FAK: iMSCs vs. iPSCs+DPBS(Ca-Mg-)	p<0.0001
		FAK: iMSCs+DPBS(Ca-Mg-) vs. iPSCs	p<0.0001
		FAK: iMSCs+DPBS(Ca-Mg-) vs. iPSCs+DPBS(Ca-Mg-)	p=0.0002
		FAK: iPSCs vs. iPSCs+DPBS(Ca-Mg-)	p=0.5067
		p-FAK/FAK: iMSCs vs. iMSCs+DPBS(Ca-Mg-)	p=0.0005
		p-FAK/FAK: iMSCs vs. iPSCs	p<0.0001
		p-FAK/FAK: iMSCs vs. iPSCs+DPBS(Ca-Mg-)	p<0.0001
		p-FAK/FAK: iMSCs+DPBS(Ca-Mg-) vs. iPSCs	p=0.0054
		p-FAK/FAK: iMSCs+DPBS(Ca-Mg-) vs. iPSCs+DPBS(Ca-Mg-)	p<0.0001
		p-FAK/FAK: iPSCs vs. iPSCs+DPBS(Ca-Mg-)	p=0.0255
**Fig. 5F**	Student's t-test	ITGA6: iPSCs vs. iMSCs	p<0.0001
	One-way ANOVA	p-FAK: iPSCs:NC vs. iPSCs:15min	p=0.9146
		p-FAK: iPSCs:NC vs. iPSCs:30min	p=0.0005
		p-FAK: iPSCs:NC vs. iPSCs:60min	p=0.0003
		p-FAK: iPSCs:NC vs. iPSCs:120min	p<0.0001
		p-FAK: iPSCs:NC vs. iMSCs:NC	p<0.0001
		p-FAK: iPSCs:30min vs. iMSCs:30min	p=0.0276
		p-FAK: iMSCs:NC vs. iMSCs:15min	p=0.0002
		p-FAK: iMSCs:NC vs. iMSCs:30min	p<0.0001

		p-FAK: iMSCs:NC vs. iMSCs:60min	p<0.0001
		p-FAK: iMSCs:NC vs. iMSCs:120min	p<0.0001
		FAK: iPSCs:NC vs. iPSCs:15min	p>0.9999
		FAK: iPSCs:NC vs. iPSCs:30min	p>0.9999
		FAK: iPSCs:NC vs. iPSCs:60min	p>0.9999
		FAK: iPSCs:NC vs. iPSCs:120min	p>0.9999
		FAK: iPSCs:NC vs. iMSCs:NC	p=0.1265
		FAK: iPSCs:30min vs. iMSCs:30min	p=0.2222
		FAK: iMSCs:NC vs. iMSCs:15min	p>0.9999
		FAK: iMSCs:NC vs. iMSCs:30min	p>0.9999
		FAK: iMSCs:NC vs. iMSCs:60min	p>0.9999
		FAK: iMSCs:NC vs. iMSCs:120min	p>0.9999
		p-FAK/FAK: iPSCs:NC vs. iPSCs:15min	p>0.9999
		p-FAK/FAK: iPSCs:NC vs. iPSCs:30min	p=0.0927
		p-FAK/FAK: iPSCs:NC vs. iPSCs:60min	p=0.0487
		p-FAK/FAK: iPSCs:NC vs. iPSCs:120min	p=0.0185
		p-FAK/FAK: iPSCs:NC vs. iMSCs:NC	p<0.0001
		p-FAK/FAK: iPSCs:30min vs. iMSCs:30min	p=0.0003
		p-FAK/FAK: iMSCs:NC vs. iMSCs:15min	p=0.0499
		p-FAK/FAK: iMSCs:NC vs. iMSCs:30min	p<0.0001
		p-FAK/FAK: iMSCs:NC vs. iMSCs:60min	p<0.0001
		p-FAK/FAK: iMSCs:NC vs. iMSCs:120min	p<0.0001
**Fig. 6C**	Student's t-test	p-FAK: iMSCs vs. iMSCs+FAKi	p<0.0001
		FAK: iMSCs vs. iMSCs+FAKi	p<0.0001
		p-FAK/FAK: iMSCs vs. iMSCs+FAKi	p<0.0001
**Fig. 6E**	Student's t-test	p-FAK: iMSCs vs. iMSCs+FAKi	p=0.0001
		FAK: iMSCs vs. iMSCs+FAKi	p=0.0002
		p-FAK/FAK: iMSCs vs. iMSCs+FAKi	p=0.0016
**Fig. 7B**	Student's t-test	OCT4: iPSCs vs. shOCT4-iPSCs	p=0.000507
		SOX2: iPSCs vs. shOCT4-iPSCs	p=0.000537
		ITGA1: iPSCs vs. shOCT4-iPSCs	p<0.000001
		ITGA2: iPSCs vs. shOCT4-iPSCs	p=0.056988
		ITGA3: iPSCs vs. shOCT4-iPSCs	p=0.263471
		ITGA5: iPSCs vs. shOCT4-iPSCs	p=0.003651
		ITGA6: iPSCs vs. shOCT4-iPSCs	p=0.012659
		ITGA7: iPSCs vs. shOCT4-iPSCs	p=0.023149

		ITGAV: iPSCs vs. shOCT4-iPSCs	p=0.000446
		ITGB1: iPSCs vs. shOCT4-iPSCs	p=0.001042
		ITGB5: iPSCs vs. shOCT4-iPSCs	p=0.000003
**Fig. 7E**	Student's t-test	p-FAK: iPSCs vs. shOCT4-iPSCs	$p<0.0001$
		FAK: iPSCs vs. shOCT4-iPSCs	$p<0.0001$
		p-FAK/FAK: iPSCs vs. shOCT4-iPSCs	$p<0.0001$
**Fig. 7G**	Student's t-test	p-FAK: iPSCs vs. shOCT4-iPSCs	p=0.0004
		FAK: iPSCs vs. shOCT4-iPSCs	p=0.0629
		p-FAK/FAK: iPSCs vs. shOCT4-iPSCs	p=0.0015
		ITGA6: iPSCs vs. shOCT4-iPSCs	p=0.0151
**Fig. 8C**	One-way ANOVA	ITGA6: iPSCs vs. shITGA6-1	$p<0.0001$
		ITGA6: iPSCs vs. shITGA6-2	$p<0.0001$
		ITGA6: iPSCs vs. shITGA6-3	p=0.0004
		OCT4: iPSCs vs. shITGA6-1	p=0.0002
		OCT4:iPSCs vs. shITGA6-2	$p<0.0001$
		OCT4:iPSCs vs. shITGA6-3	p=0.0155
**Fig. 8E**	One-way ANOVA	p-FAK: iPSCs vs. shITGA6-1	p=0.005
		p-FAK: iPSCs vs. shITGA6-2	p=0.0067
		p-FAK: iPSCs vs. shITGA6-3	p=0.0336
		FAK: iPSCs vs. shITGA6-1	p=0.4296
		FAK:iPSCs vs. shITGA6-2	p=0.5666
		FAK:iPSCs vs. shITGA6-3	$p>0.9999$
		p-FAK/FAK: iPSCs vs. shITGA6-1	p=0.0007
		p-FAK/FAK: iPSCs vs. shITGA6-2	p=0.0008
		p-FAK/FAK: iPSCs vs. shITGA6-3	p=0.0007
		ITGA6: iPSCs vs. shITGA6-1	p=0.005
		ITGA6: iPSCs vs. shITGA6-2	p=0.0045
		ITGA6: iPSCs vs. shITGA6-3	p=0.0191
**Fig. 8G**	One-way ANOVA	p-FAK: iPSCs vs. shITGA6-1	$p<0.0001$
		p-FAK: iPSCs vs. shITGA6-2	$p<0.0001$
		p-FAK: iPSCs vs. shITGA6-3	$p<0.0001$
		FAK: iPSCs vs. shITGA6-1	$p<0.0001$
		FAK:iPSCs vs. shITGA6-2	$p<0.0001$
		FAK:iPSCs vs. shITGA6-3	$p<0.0001$
		p-FAK/FAK: iPSCs vs. shITGA6-1	$p<0.0001$
		p-FAK/FAK: iPSCs vs. shITGA6-2	$p<0.0001$

		p-FAK/FAK: iPSCs vs. shITGA6-3	p<0.0001
**Fig. 8L**	Student's t-test	p-FAK: iPSCs vs. ITGA6-blocked iPSCs	p=0.0002
		FAK: iPSCs vs. ITGA6-blocked iPSCs	p=0.7572
		p-FAK/FAK: iPSCs vs. ITGA6-blocked iPSCs	p=0.0005
**Supplemental Fig. S1B**	One-way ANOVA	OCT4: EPSCs-006-1 vs. HUVECs	p<0.0001
		OCT4: EPSCs-001-5 vs. HUVECs	p<0.0001
		OCT4: iPSCs-006-1 vs. HUVECs	p<0.0001
		OCT4: iPSCs-001-5 vs. HUVECs	p<0.0001
		SOX2: EPSCs-006-1 vs. HUVECs	p<0.0001
		SOX2: EPSCs-001-5 vs. HUVECs	p<0.0001
		SOX2: iPSCs-006-1 vs. HUVECs	p=0.0013
		SOX2: iPSCs-001-5 vs. HUVECs	p<0.0001
		Nanog: EPSCs-006-1 vs. HUVECs	p=0.0009
		Nanog: EPSCs-001-5 vs. HUVECs	p=0.0002
		Nanog: iPSCs-006-1 vs. HUVECs	p=0.0075
		Nanog: iPSCs-001-5 vs. HUVECs	p=0.0002
		Klf17: EPSCs-006-1 vs. HUVECs	p<0.0001
		Klf17: EPSCs-001-5 vs. HUVECs	p<0.0001
		Klf17: iPSCs-006-1 vs. HUVECs	p=0.0002
		Klf17: iPSCs-001-5 vs. HUVECs	p<0.0001
		Dppa3: EPSCs-006-1 vs. HUVECs	p<0.0001
		Dppa3: EPSCs-001-5 vs. HUVECs	p<0.0001
		Dppa3: iPSCs-006-1 vs. HUVECs	p<0.0001
		Dppa3: iPSCs-001-5 vs. HUVECs	p<0.0001
**Supplemental Fig. S1J**	Student's t-test	DLX3: iPSCs vs. iPSCs-Ebs	p=0.001899
		PAX6: iPSCs vs. iPSCs-Ebs	p=0.000166
		Brachyury: iPSCs vs. iPSCs-Ebs	p=0.014996
		SOX17: iPSCs vs. iPSCs-Ebs	p=0.00011
		OCT4: iPSCs vs. iPSCs-Ebs	p=0.0003
		SOX2: iPSCs vs. iPSCs-Ebs	p=0.000235
**Supplemental Figure S4I**	Two-way ANOVA	3h:+DPBS(Ca-Mg-) vs. 3h:-DPBS(Ca-Mg-)	p>0.9999
		6h:+DPBS(Ca-Mg-) vs. 6h:-DPBS(Ca-Mg-)	p>0.9999
		24h:+DPBS(Ca-Mg-) vs. 24h:-DPBS(Ca-Mg-)	p>0.9999
**Supplemental Figure S4K**	Student's t-test	iMSCs-DPBS (Ca-Mg-) vs. iMSCs+DPBS (Ca-Mg-)	p=0.4406

**Supplemental Figure S4L**	Two-way ANOVA	0h:-DPBS (Ca-Mg-) vs. 0h:+DPBS (Ca-Mg-)	p>0.9999
		24h:-DPBS (Ca-Mg-) vs. 24h:+DPBS (Ca-Mg-)	p>0.9999
		48h:-DPBS (Ca-Mg-) vs. 48h:+DPBS (Ca-Mg-)	p>0.9999
		72h:-DPBS (Ca-Mg-) vs. 72h:+DPBS (Ca-Mg-)	p>0.9999
**Supplemental Figure S6B**	Student's t-test	PAX6: iPSCs vs. Neuroectoderm	p=0.000418
		Brachyury: iPSCs vs. Neuroectoderm	p<0.000001
		SOX17: iPSCs vs. Neuroectoderm	p=0.527345
		OCT4: iPSCs vs. Neuroectoderm	p=0.000033
		ITGA6: iPSCs vs. Neuroectoderm	p=0.0017
		PAX6: iPSCs vs. Mesendoderm	p<0.000001
		Brachyury: iPSCs vs. Mesendoderm	p=0.000265
		SOX17: iPSCs vs. Mesendoderm	p=0.00068
		OCT4: iPSCs vs. Mesendoderm	p=0.000002
		ITGA6: iPSCs vs. Mesendoderm	p=0.000051
**Supplemental Figure S6F**	Two-way ANOVA	pFAK: iPSCs vs. Neuroectoderm	p<0.0001
		pFAK: iPSCs vs. Mesendoderm	p=0.0009
		FAK: iPSCs vs. Neuroectoderm	p<0.0001
		FAK: iPSCs vs. Mesendoderm	p>0.9999
		p-FAK/FAK: iPSCs vs. Neuroectoderm	p<0.0001
		p-FAK/FAK: iPSCs vs. Mesendoderm	p=0.0018
**Supplemental Figure S6H**	Two-way ANOVA	pFAK: iPSCs vs. Neuroectoderm	p=0.0076
		pFAK: iPSCs vs. Mesendoderm	p=0.0006
		FAK: iPSCs vs. Neuroectoderm	p>0.9999
		FAK: iPSCs vs. Mesendoderm	p=0.6667
		p-FAK/FAK: iPSCs vs. Neuroectoderm	p=0.0432
		p-FAK/FAK: iPSCs vs. Mesendoderm	p=0.0132
		ITGA6: iPSCs vs. Neuroectoderm	p<0.0001
		ITGA6: iPSCs vs. Mesendoderm	p=0.0005
**Supplemental Figure S7B**	One-way ANOVA	iPSCs vs. iPSCs+10 nM FAKi	p=0.9923
		iPSCs vs. iPSCs+100 nM FAKi	p=0.0028
		iPSCs vs. iPSCs+1 uM FAKi	p=0.0478

**Supplemental Figure S7C**	Student's t-test	iPSCs vs. iPSCs-FAKi	p<0.0001

Table S3. Statistical methods and p-values used in this study
